# Overexpression of the Catalytically Impaired Taspase1^T234V^ or Taspase1^D233A^ Variants Does Not Have a Dominant Negative Effect in T(4;11) Leukemia Cells

**DOI:** 10.1371/journal.pone.0034142

**Published:** 2012-05-03

**Authors:** Carolin Bier, Rouven Hecht, Lena Kunst, Sabine Scheiding, Désirée Wünsch, Dorothée Goesswein, Günter Schneider, Oliver H. Krämer, Shirley K. Knauer, Roland H. Stauber

**Affiliations:** 1 Molecular and Cellular Oncology, Mainz Screening Center (MSC), University Hospital of Mainz, Mainz, Germany; 2 Institute for Molecular Biology, Center for Medical Biotechnology (ZMB), University Duisburg-Essen, Essen, Germany; 3 Institute for Biochemistry and Biophysics/Centre for Molecular Biomedicine (CMB), Friedrich-Schiller-University Jena, Jena, Germany; 4 Department of Internal Medicine II, Technical University of Munich, Munich, Germany; Stanford University, United States of America

## Abstract

**Background:**

The chromosomal translocation t(4;11)(q21;q23) is associated with high-risk acute lymphoblastic leukemia of infants. The resulting AF4•MLL oncoprotein becomes activated by Taspase1 hydrolysis and is considered to promote oncogenic transcriptional activation. Hence, Taspase1’s proteolytic activity is a critical step in AF4•MLL pathophysiology. The Taspase1 proenzyme is autoproteolytically processed in its subunits and is assumed to assemble into an αββα-heterodimer, the active protease. Therefore, we investigated here whether overexpression of catalytically inactive Taspase1 variants are able to interfere with the proteolytic activity of the wild type enzyme in AF4•MLL model systems.

**Methodology/Findings:**

The consequences of overexpressing the catalytically dead Taspase1 mutant, Taspase1^T234V^, or the highly attenuated variant, Taspase1^D233A^, on Taspase1’s processing of AF4•MLL and of other Taspase1 targets was analyzed in living cancer cells employing an optimized cell-based assay. Notably, even a nine-fold overexpression of the respective Taspase1 mutants neither inhibited Taspase1’s *cis*- nor *trans*-cleavage activity *in vivo.* Likewise, enforced expression of the α- or β-subunits showed no *trans*-dominant effect against the ectopically or endogenously expressed enzyme. Notably, co-expression of the individual α- and β-subunits did not result in their assembly into an enzymatically active protease complex. Probing Taspase1 multimerization in living cells by a translocation-based protein interaction assay as well as by biochemical methods indicated that the inactive Taspase1 failed to assemble into stable heterocomplexes with the wild type enzyme.

**Conclusions:**

Collectively, our results demonstrate that inefficient heterodimerization appears to be the mechanism by which inactive Taspase1 variants fail to inhibit wild type Taspase1’s activity in *trans*. Our work favours strategies targeting Taspase1’s catalytic activity rather than attempts to block the formation of active Taspase1 dimers to interfere with the pathobiological function of AF4•MLL.

## Introduction

Chromosomal rearrangements of the mixed lineage leukemia (*MLL*) gene with numerous partner genes are frequently found in acute myeloid (AML) and acute lymphoblastic leukemia (ALL) [1], [2]. Fused on chromosome4 (AF4) is the most common translocation partner in MLL-mediated leukemia, resulting in the expression of the AF4•MLL and MLL•AF4 fusion proteins. Although the pathomechanism of t(4;11)-mediated leukemia is still discussed controversially, expression of the AF4•MLL fusion enhanced the hematopoietic repopulating potential of CD34^+^ cells, and led to the development of predominantly proB ALL in a mouse model [2], [3]. Similar to the prototypic MLL protein, the AF4•MLL fusion protein contains cleavage-sites for Threonine Aspartase 1 (Taspase1, Tasp) and, is considered a bona fide substrate for this protease [4], [5], [6], [7], [8].

In leukemic cells, the unprocessed AF4•MLL fusion protein is a substrate for the E3-ubiquitin ligases SIAH1 and SIAH2 [9], [10]. SIAH1 and SIAH2, belonging to the family of the seven in absentia homolog (SIAH), are E3 ligases involved in ubiquitination and proteasome-mediated degradation of specific proteins [9], [10]. Therefore the presence of these ligases leads to a low steady-state level of the AF4•MLL fusion, hampering its detection and experimental analysis. However, following its proteolytic processing by Taspase1, the AF4•MLL cleavage products AF4•MLL.N and MLL.C heterodimerize through their FYRN and FYRC interaction domains, forming a high molecular-weight protein complex resistant to SIAH-mediated degradation [9], [10]. Thus, chemico-genetic interference with Taspase1’s activity is expected to promote AF4•MLL degradation, thereby precluding the activation of oncogenic programs and disease development. Moreover, a total of at least 64 MLL chromosomal-fusion partners have been characterized so far at the molecular level, associated with disease (**Table S1**) [1], [11], [12]. Hence, Taspase1’s proteolytic processing of MLL fusions in general may contribute to various pathologies.

The human Taspase1 gene encodes a protein of 420 amino acids (aa) and is able to cleave other substrates in *trans* by recognizing a conserved peptide motif (Q^3^[F,I,L,V]^2^D^1^↓G^1’^x^2’^D^3’^D^4’^) with an aspartate at the P1 position [6], [7]. The discovery of Taspase1 founded a new class of endopeptidases that utilize the N-terminal threonine of its mature β-subunit as the active site, which is generated by autoproteolysis of the proenzyme (*cis*-cleavage) [6]. Mutation of the catalytic nucleophile, Thr^234^, completely abolishes Taspase1’s catalytic activity [6], [13]. Based on data mainly derived from analyzing bacterially expressed Taspase1, it is assumed that the proenzyme assembles into an asymmetric αββα-heterodimer following autoproteolysis, representing the active protease [6], [13].

Protein-protein interactions (PPIs) in general are key players for multiple (patho)biological cellular processes [14]. Thus, interfering with disease-relevant interactomes *via* enforced expression of dominant-negative mutants and/or small molecules has emerged as a promising, though challenging strategy for human therapeutics [15], [16]. Blocking the p53-mdm2 interaction with synthetic molecules had been shown to induce p53 activation and thereof tumor cell death [17]. Likewise, the peptide-mediated disruption of the AF4–AF9 protein complex, or targeting the oligomerization domain of RUNX1/ETO interfered with the activity of the fusion proteins in leukemic cells [18], [19]. Recently, we also showed that it is in principle possible to specifically inhibit and to destroy the AF4•MLL oncoprotein by genetic PPIs inhibitors [9].

Consequently, as currently no effective synthetic Taspase1 inhibitors are available, we here investigated whether selectively interfering with the formation of the proposed active Taspase1 αββα-heterodimer would block processing of the AF4•MLL fusion and thus, may prevent leukemogenesis [20], [21]. Studies investigating the biological consequences of overexpressing inactive Taspase1 variants have not been performed before. Hence we here developed and employed novel cell-based assays allowing to functionally monitor the effects of overexpressing catalytically inactive or attenuated Taspase1 mutants on Taspase1’s processing of AF4•MLL in living cancer cells in real time. To our knowledge, this is the first comprehensive study addressing Taspase1 multimerization and genetic interference thereof *in vivo*.

## Materials and Methods

### Antibodies (Ab), Reagents and Compounds

Ab used: anti-(α)-GapDH (Glyceraldehyde-3-phosphate dehydrogenase, sc-47724; Santa Cruz Biotechnology, Heidelberg, Germany); α-GFP (green fluorescent protein, sc-8334; Santa Cruz Biotechnology, Heidelberg, Germany); α-GST (glutathione S-transferase, sc-57753; Santa Cruz Biotechnology, Heidelberg, Germany); α-NPM1 (nucleophosmin, #3542 Cell Signaling); α-Tasp_C_ (directed against the C-terminus of Taspase1, AP1330b BioCat GmbH, Heidelberg, Germany); α-Tasp_N_ (directed against the N-terminus of Taspase1, sc-85945; Santa Cruz Biotechnology, Heidelberg, Germany). Appropriate HRP-conjugated secondary antibodies (Santa Cruz Biotechnology, Heidelberg, Germany) were used.

### Plasmids

AF4•MLL, transcription factor IIA (TFIIA) and upstream stimulatory factor2 (USF2) indicator protein expression plasmids were derived from pNLS-GFP/GST-CS3-RevNES (pCasp3-Clev), encoding a fusion composed of the SV40 large T-antigen nuclear localization signal (NLS), GST, GFP, the Caspase3 cleavage site (CS3), and a strong nuclear export signal (NES) of the human immunodeficiency virus type 1 (HIV-1) Rev protein. [7], [22] In p_NLS-GFP/GST-AF4•MLL_S1/2-NES_Rev_ (pA•M_S1/2), p_NLS-GFP/GST-AF4•MLL_S1-NES_Rev_ (pA•M_S1) and p_NLS-GFP/GST-AF4•MLL_S2-NES_Rev_ (pA•M_S2), CS3 was replaced by the Taspase1 cleavage site from AF4•MLL (S1/2 – both cleavage sites aa 1582–1710; S1 – first cleavage site: ^1600^AEGQVDGADD^1609^; S2 – second cleavage site:^ 1652^KISQLDGVDD^1661^), as well as by a GSGS linker following the cleavage site. To determine the linker sequences allowing optimal processing by Taspase1, plasmids containing the sequences ^1655^QLDGVDD^1661^, GSGS^1655^QLDGVDD^1661^ or^ 1652^KISQLDGVDD^1661^G were established likewise. pA•M_S1_mut_ and pA•M_S2_mut_ encode fusions containing mutated Taspase1 cleavage sites, in which P1 and P1’ were exchanged by alanin (S1_mut_: ^1600^AEGQVAAADD^1609^ and S2_mut_: ^1652^KISQLAAVDD^1661^), precluding their processing by Taspase1. pTFIIA_S, pUSF2_S, pTFIIA-GFP and pUSF2-GFP were described. [7] Besides the plasmids expressing green fluorescent protein fusions, we also constructed versions in which GFP was replaced by the red-fluorescent protein mCherry or RFP allowing performing dual- or triple-color *in vivo* assays.

Expression constructs encoding untagged Taspase1, Taspase1 fusion with autofluorescent proteins, including the red-fluorescent protein mCherry, a cytoplasmatic version of GFP-tagged Taspase1 (pTasp_cyt_), and NPM1 as untagged or fusions with autofluorescent proteins were described. [7], [23] Expression constructs encoding Taspase1 as a fusion with the small HA-tag was generated as described. [9], [24] The Taspase1 expression plasmid was used as template to amplify the Taspase1 α- and β-subunits. Cloning of the subunits into expression vectors pc3-GFP and pc3-BFP using BamHI/NheI-restriction sites, respectively, allowed the expression of Taspase1 subunits as fusions with green and blue fluorescent proteins as described. [25] A cytoplasmatic version of a GFP-tagged Taspase1 β-subunit is encoded by pTasp-β_Cyt_, which was described. [20] Plasmids pTasp^T234V^- and pTasp^D233A^-GFP/-mCherry/-BFP or their untagged versions were generated by splice overlap extension PCR as reported. [7] pRevM10BL-RFP was generated by replacing BFP by RFP in RevM10BL-BFP using NheI- and EcoRI-restriction sites. pF143 encoding GFP, F145 encoding BFP and pBluescript (BSK) were described. [23], [26] Bacterial expression plasmids pGEX_GST-Tasp-GFP and pGEX_GST-GFP were described. [20] A detailed overview of plasmids and oligonucleotides used for PCR amplification and cloning can be found in **Table S2** and **S3**.

### Cells, Transfection and Microscopy

Leukemic and solid cancer cell lines used in the study were maintained and transfected as described [9], [23]. Observation and image analysis of living or fixed cells were performed as described [7], [27]. To determine the average intracellular protein localization, at least 200 fluorescent cells from three separate images were examined in three independent experiments, and representative images are shown. The number of cells exhibiting cytoplasmic (C; cytoplasmic signal >80% of the total cellular signal), cytoplasmic and nuclear (C/N), or nuclear (N; nuclear signal >80% of the total cellular signal) fluorescence was counted. As standards for this semiquantitative determination, the total cellular BFP (blue), GFP (green) or mCherry/RFP (red) signal was quantitated by calculating the integrated pixel intensity in the imaged cell multiplied by the area of the cell in 100 fluorescent cells using a digital AxioCam CCD camera (Carl Zeiss, Jena, Germany) as described [7], [27]. The nuclear signal was similarly obtained by measuring the pixel intensity in the respective nuclei. Nuclei were marked by Hoechst 33258 staining as described [20], [27].

Criteria for efficient in vivo protein interaction using the protein interaction assay was that in >80% of 200 mCherry- and GFP-positive cells, mCherry and GFP co-localized at the nucleolus. Co-localization was further quantitated by confocal laser scanning microscopy using the “Overlap coefficient according to Manders” co-localization algorithm as described [24], [27]. Colocalization coefficients represent “Overlap coefficient according to Manders” [9], [24], [27]. 
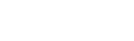
 *R* represents the calculated overlap coefficient, *S1* and *S2* the measured signal in the two channels, and *i* a specific pixel of the taken image. Therefore, the calculated *R* value indicates an overlap of the signals and thus represents the true degree of colocalization. Other presented coefficients resembling Pearson’s correlation coefficient *R_r_*. Overlap coefficients *k_1_* and *k_2_* and colocalization coefficients *m_1_* and *m_2_* were described [27], [28].

### Protein Extraction and Immunoblot Analysis

Preparation of whole lysates from cells and immunoblotting were carried out as described [23]. Equal loading of lysates was controlled by reprobing blots for GapDH as described [7].

### Gel Filtration Chromatography

MV4;11 cells were harvested by centrifugation at 350°g, 4°C, 5°min, washed with ice-cold phosphate-buffered saline and lysed by the addition of NETN buffer (100 mM NaCl; 10 mM Tris, pH 8; 10% Glycerol; 1 mM EDTA; 0.5% v/v NP40; 1 mM DTT; 1 mM PMSF; 1 fold Complete Protease Inhibitor – Roche, Germany) as described in [7]. After sonication lysates were centrifuged (14.000 rpm, 4°C, 30 min) and supernatant filtered (micro-centrifuge-filtered tubes 0.2 µm – Laborservice Onken GmbH) prior to gel filtration chromatography. The extracts were fractionated using size exclusion chromatography with Superose-6 10/300 GL columns (GE Healthcare FPLC system, optimal separation range from 3 MDa to 5 kDa). For system calibration purified aprotinin (6.5 kDa), ribunclease A (13.7 kDa), carbonic anhydrase (29 kDa) and ovalbumin (44 kDa) were purchased from Sigma Aldrich (Sigma Aldrich, Munich, Germany) and used as standards. Loading and elution of the FPLC system was carried out in 50 mM Phosphate with 0.15 M NaCl pH 7. 500 µl fractions were collected and stored at −70°C until analyzed by SDS-PAGE as described [29].

### Immunoprecipitation (IP)

IP of GFP-tagged proteins was performed using α-GFP magnetic beads and μ-MACS columns (MiltenyiBiotec, Bergisch Gladbach, Germany) [7], [20]. Briefly, whole cell lysates were incubated with 50 µl α-GFP magnetic beads for 30 min on ice. Lysates with magnetic beads were applied onto the columns, washed, 20 µl elution buffer was applied and incubated for 5 min. To complete protein elution, 50 µl elution buffer were applied. 30 µl of the eluate, as well as 3% of input were analyzed as outlined [23].

### Statistical Analysis

For experiments stating *p*-values, a paired Student’s t-test was performed. Unless stated otherwise, *p*-values represent data obtained from three independent experiments done in triplicate. *p-*values <0.05 were considered significant [23].

## Results

### Monitoring Taspase1 Processing of AF4•MLL Substrates in Living Cells

**Figure 1:**
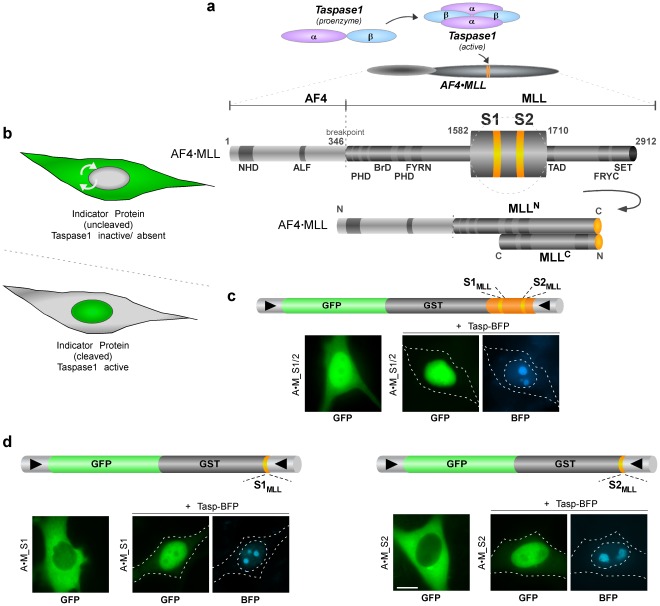
Analyzing Taspase1’s processing of AF4•MLL substrates in living cells. **A.** Autoproteolysis of the Taspase1 proenzyme is assumed to trigger formation of the active αββα-heterodimer, which hydrolyses the AF4**•**MLL fusion protein. Following processing, the cleavage products AF4**•**MLL.N and MLL.C heterodimerize, forming a high molecular-weight protein complex resistant to degradation. Domain organization of the AF4**•**MLL fusion. Taspase1 cleavage sites, S1 (QVDGADD) and S2 (QLDGVDD), are highlighted. NHD: N-terminal homology domain; ALF: AF4/LAF4/FMR2 homology domain; PHD: plant homeodomain; BrD: bromodomain; FRYN: F/Y rich domain N-terminal; TAD: transactivation domain; FRYC: F/Y rich domain C-terminal; SET: suppressor of variegation, enhancer of zeste and trithorax. Domains are not drawn to scale. **B.** Principle of the cell-based biosensor assay to analyze Taspase1-mediated AF4**•**MLL processing. The indicator protein localizes predominantly to the cytoplasm but is continuously shuttling between the nucleus and the cytoplasm. Co-expression of active Taspase1 results in the proteolytic removal of the NES, thereby triggering nuclear accumulation of the green fluorescent indicator. **C–D.** Domains of the indicator protein, composed of GST, GFP, combinations of a nuclear import (?: NLS) and an export (?: NES) signal, combined with the indicated cleavage sites of AF4**•**MLL. **c.** A**•**M_S1/2 containing both cleavage sites is already partially processed by endogenous Taspase1 (left panel), but is completely nuclear upon expression of Taspase1-BFP (right panel). **D.** Indicator proteins containing only one cleavage site (A**•**M_S1 or A **•**M_S2) are cytoplasmic in their uncleaved state, whereas ectopic expression of active Taspase1 triggers their cleavage and complete nuclear accumulation. GFP/BFP were visualized by fluorescence microscopy in living HeLa transfectants 24 h after transfection. Scale bars, 10 µm. Dashed lines mark cytoplasmic/nuclear cell boundaries obtained from the corresponding phase contrast images.

As the AF4•MLL fusion is a substrate for SIAH1/SIAH2, its steady-state level is low, thereby hampering its detection and experimental analysis [8]. In fact, studies visualizing the intracellular localization of uncleaved or processed AF4•MLL protein are still missing [3]. Also, as biochemical data and *in vitro* interference strategies must be effective at the cellular level, they have to be verified *in vivo*. Hence, we set out to establish a suitable cell-based assay in the most relevant test tube, the living cell (**Figure 1**).

To analyze Taspase1’s processing of AF4•MLL substrates in living cells, we exploited a two component autofluorescent indicator protein system [22]. Therefore, the AF4•MLL residues surrounding the two Taspase1 cleavage sites (A•M_S1/2: aa 1582–1710 of the AF4•MLL fusion protein) were inserted into a backbone composed of GST, GFP, a N-terminal nuclear import (NLS) and a C-terminal nuclear export signal (NES) (**Figure 1a**). As second element the Taspase1 open reading frame was cloned from the Taspase1-expressing acute monocytic leukemia (AMoL) cell lines MV4;11 and THP-1, carrying a t(4;11) or t(9;11) translocation, respectively. Both sequences are identical to the one described by Hsieh et al. [6]. The rationale of this specific assay set-up was that the resulting NLS-GFP/GST-AF4•MLL_S1/2-NES fusion protein (A•M_S1/2) localizes predominantly to the cytoplasm, whereas Taspase1-mediated cleavage liberates the NES triggering nuclear accumulation (**Figure 1b**). However, due to the presence of two cleavage-sites, the A•M_S1/2 indicator protein was already (partially) cleaved by endogenous Taspase1 (**Figure 1c**), which was especially evident in cell lines with high protease expression levels (**Figure S1a**).

As this efficient processing precludes the use of the indicator protein in its current set-up, we engineered proteins harboring only individual AF4•MLL cleavage sites, A•M_S1 (^1600^AEGQVDGADD^1609^) or A•M_S2 (^1652^KISQLDGVDD^1661^), which are expected to be less efficiently processed. As shown in **Figure S1b–e**, we found that the addition of a linker sequence was crucial for the performance of the A•M_S1 and A•M_S2 indicator proteins. Both indicator proteins localize predominantly to the cytoplasm in cancer cells, whereas ectopic expression of biologically active Taspase1 promoted their cleavage and complete nuclear accumulation (**Figure 1d**). As a control, constructs containing non-functional Taspase1 cleavage sites (A•M_S1_mut_, aa ^1600^AEGQVAAADD^1609^ or A•M_S2_mut_,^ 1652^KISQLAAVDD^1661^) remained cytoplasmic (data not shown).

**Figure 2:**
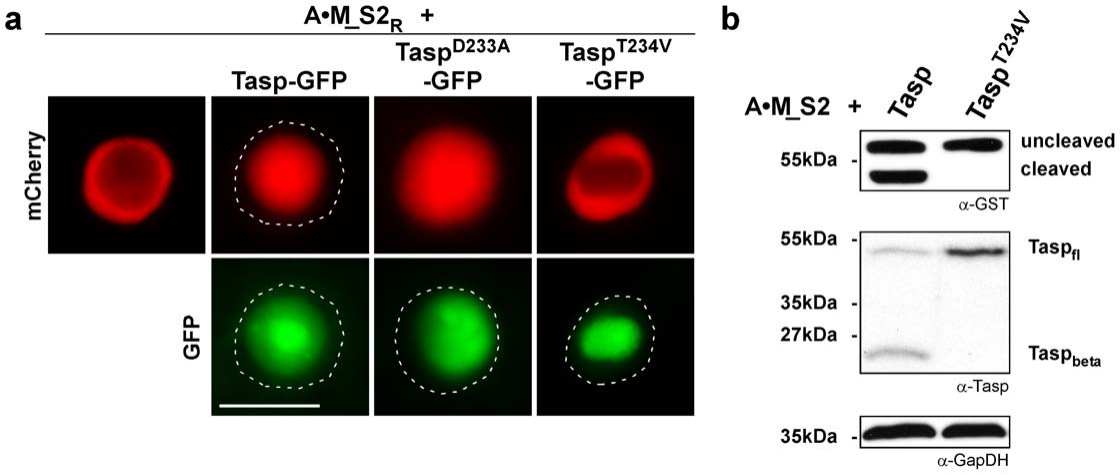
Activity and complex formation of Taspase1 and catalytically inactive mutants. A. Taspase1 processing of AF4**•**MLL substrates in leukemic cells. Co-transfection of Tasp-GFP resulted in proteolytic cleavage and nuclear accumulation of the red fluorescent biosensor, A**•**M_S2_R_, in K562 cells. In contrast, co-expression of Tasp^D233A^-GFP leads to partial processing and nuclear translocation, while Tasp^T234V^-GFP was completely inactive. Localization was analyzed 24 h post transfection. GFP/mCherry were visualized by fluorescence microscopy. Scale bars, 10 µm. **B.** Processing of AF4**•**MLL substrates. Co-transfection of Tasp resulted in proteolytic cleavage of the biosensor A**•**M_S2_R_ in 293T cells as indicated by immunoblot. In contrast, Tasp^T234V^ was inactive in *cis* and *trans*. Proteins were visualized using α-GST or α-Taspase1 Abs. GapDH served as loading control. fl, unprocessed Taspase1; Tasp_β_, Taspase1 β-subunit.

Also in leukemic cells Taspase1 localizes predominantly to cellular nucleus and both indicator proteins localize predominantly to the cytoplasm, while co-expression of either indicator protein and the active protease promotes the indicator protein’s nuclear accumulation (**Figure 2a**). Mutation of Thr^234^ into Val (Tasp^T234V^) or Asp^233^ into Ala (Tasp^D233A^) of Taspase1 affected autoprocessing as well as the protease’s *trans*-cleavage activity. Both mutants showed a nuclear but not nucleolar localization, but in contrast to the wild type protease co-expression of the catalytically inactive Tasp^T234V^- or Tasp^D233A^-GFP mutants did not result in effective cleavage and nuclear translocation of the indicator proteins. Similar results were obtained upon co-expression of untagged Tasp^T234V^ or Tasp^D233A^ as well as of their fusions to the red- (mCherry) or blue-fluorescent (BFP) protein, allowing their independent detection in living cells (**Figure 2a**, **Figure S2** and **Table S4**), which was confirmed by immunoblot analysis (**Figure 2b**) [6], [13].

These results demonstrate the specificity of the assay system for Taspase1 and underline the advantage of using rational combinations of multi-color autofluorescent proteins to study Taspase1’s biological activity in living cells. Interestingly, we found that Tasp^D233A^ showed cleavage-site specificity, being able to process A•M_S2_R_, albeit with a highly attenuated activity, but not A•M_S1_R_ (**Figure S2**). Although fusions of Taspase1 with autofluorescent proteins have been shown to be fully functional, we confirmed these results by employing untagged or HA-tagged Taspase1 variants (data not shown) [23]. Hence, our system is also applicable to assess Taspase1 *trans*-cleavage activity on the individual AF4•MLL cleavage sites independently from each other.

### Targeting Taspase1 Function in Trans by Catalytically Inactive Mutants

**Figure 3:**
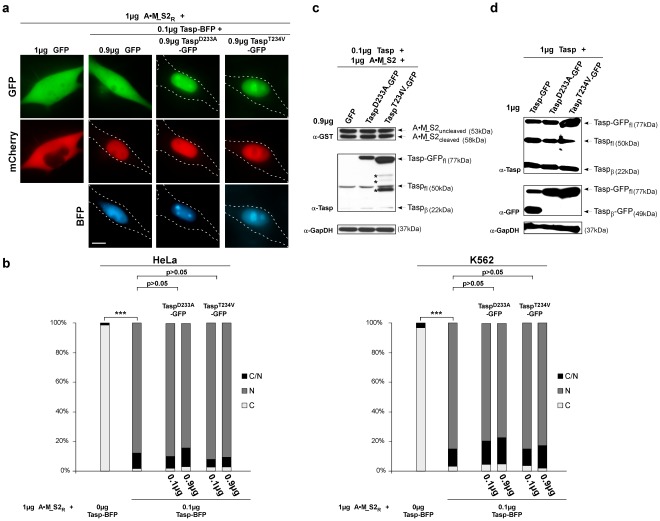
Overexpression of inactive Taspase1 mutants does not inhibit Taspase1’s *cis-* or *trans*-cleavage activity. **A.** Cells were transfected with 1 µg of A**•**M_S2_R_, 0.1 µg Tasp-BFP together with the indicated amounts of inactive Taspase1 mutants or GFP expression plasmid, and analyzed 24 h later. Even co-transfection of a nine-fold excess of plasmids encoding the inactive Taspase1 variants did not affect A**•**M_S2_R_ processing in living HeLa cells. **B.** The number of HeLa (left panel) or leukemic K562 cells (right panel) showing cytoplasmic (C), cytoplasmic and nuclear (N/C) or nuclear (N) fluorescence was counted in at least 200 A**•**M_S2_R_-expressing cells. Results from one representative experiment of each indicated cell line are shown. Whereas the number of cell displaying cytoplasmic fluorescence significantly decreased by *trans*-cleavage upon co-transfection of 0.1 µg Tasp-BFP expression plasmid (***: p<0.0001), no significant *trans*-dominant negative effect was evident for Taspase1 mutants. **C.** Taspase1 *trans*-cleavage of A**•**M_S2_R_ is unaffected by inactive Taspase1 mutants as shown by immunoblot analysis of 293T cells transfected with the indicated expression plasmids. Proteins and cleavage products were visualized using α-GST and α-Tasp Ab. GapDH served as loading control. **D.** *Cis*-cleavage of Taspase1 is not inhibited by inactive Taspase1 mutants as shown by immunoblot analysis of 293T cells transfected with 1 µg of the indicated expression plasmids.

Subsequently, we used the established bioassay to investigate the consequences of overexpressing catalytically impaired Taspase1 mutants on the activity of the wild type (WT) enzyme *in trans*. We reasoned if inactive Taspase1 mutants are capable of forming heterodimers with WT Taspase1 (heterodimerization model), enforced overexpression of these mutants should have a dominant-negative effect. Besides the catalytically dead Tasp^T234V^-GFP mutant, we also included Tasp^D233A^-GFP in the analysis, as this variant exists in a biologically active though highly attenuated conformation. Notably, our assay demonstrated that even co-transfecting a nine-fold excess of the Tasp^T234V^- or of the Tasp^D233A^-GFP mutants over the WT Taspase1 expression plasmid did not affect Taspase1’s processing of either the first or the second AF4•MLL cleavage site in solid as well as in leukemic cancer cell lines. These results could be independently verified in several solid as well as leukemic cancer cell lines (**Figure 3a** **/b** and **Table 1**). Immunoblot analysis confirmed that the mutants were efficiently overexpressed (**Figure 3c**). Similar results were obtained when using HA-tagged or untagged Taspase mutants (data not shown). To further exclude the formal possibility that our results are only valid for ectopically expressed Taspase1, we used the SaOs and SW480 cell lines expressing high levels of endogenous Taspase1 [23]. Upon expression in these cells, the A•M_S1/2 indicator protein is already fully or partially cleaved by endogenous Taspase1 resulting in its predominant nuclear localization (**Figure S1a** and **Table 1**). As expected, overexpression of the inactive Taspase1 variants did not inhibit the endogenous enzyme and thus, did not affect cleavage of the indicator protein in *trans* (**Table 1**).

**Table 1:** Effects of overexpressing inactive Taspase1 mutants in trans on Taspase1’s processing of various target proteins.

1 µg indicator +	0.1 µg BFP+ 0.9 µg GFP		0.1 µg Tasp-BFP+ 0.9 µg GFP		0.1 µg Tasp-BFP+ 0.9 µg Tasp^T234V^-GFP		0.1 µg Tasp-BFP+ 0.9 µg Tasp^D233A^-GFP	
localization	C	N	C	N	C	N	C	N
**A•M_S1_R_ (HeLa)**	>90%	<1%	<5%	>80%	<5%	>80%	<5%	>80%
**A•M_S1_R_ (K652)**	>85%	<1%	<7%	>75%	<7%	>75%	<7%	>75%
**A•M_S2_R_ (HeLa)**	>90%	<1%	<5%	>80%	<5%	>80%	<5%	>80%
**A•M_S2_R_ (K562)**	>85%	<1%	<5%	>80%	<5%	>80%	<5%	>80%
**TFIIA_S_R_ (HeLa)**	>90%	<1%	<5%	>80%	<5%	>80%	<5%	>80%
**USF2_S_R_ (HeLa)**	>90%	<1%	<5%	>80%	<5%	>80%	<5%	>80%

	1 µg BFP		1 µg Tasp^T234V^-BFP		1 µg Tasp^D233A^-BFP	
	C	N	C	N	C	N
**A•M_S1/2 (SaOs)**	<2%	>90%	<2%	>90%	<2%	>90%
**A•M_S1/2 (SW480)**	<5%	>80%	<5%	>80%	<5%	>80%

Leukemic (K562) and solid tumor cells were transfected with the indicated amounts of the different indicator plasmids, together with respective control plasmids, or expression plasmids encoding active or inactive Taspase1 mutants, and analyzed 24 h later. The number of cells showing cytoplasmic (C) or nuclear (N) fluorescence was counted in at least 200 indicator protein-expressing cells. Results from one representative experiment are shown. Whereas the number of transfectants displaying cytoplasmic fluorescence, i.e., uncleaved indicator protein, significantly decreased upon co-transfection of 0.1 µg Tasp-BFP expression plasmid (***: p<0.0001), no inhibition of cleavage was observed even upon co-transfection of 0.9 µg expression plasmids encoding for the inactive Taspase1 mutants.

In transfectants with high (SaOs) or intermediate (SW480) levels of endogenous Taspase1, the A•M_S1/2 indicator protein (0.2 µg expression plasmid) is already fully or partially cleaved in absence of ectopically expressed protease resulting in its predominant nuclear localization. A similar localization was observed upon co-expression of the inactive Taspase1 variants (1 µg expression plasmid), indicating that the activity of endogenous Taspase1 is not inhibited in *trans*.

Next, we further analyzed whether *cis*-cleavage of WT Taspase1 could be affected *in trans*. As shown in **Figure 3d**, co-transfection of the WT protease with GFP-tagged or untagged mutants did not inhibit Taspase1’s *cis*-cleavage activity, since the processed Taspase1 β-subunit was detectable in all plasmid combinations used. Immunoblot analysis verified that the Tasp^T234V^- or Tasp^D233A^-GFP proenzymes are impaired in their activation by autoproteolytic *cis*-cleavage (**Figure 3d**).

Also, we tested whether overexpression of the individual Taspase1 α- or β-subunit, which are clearly proteolytically inactive, affects Taspase1’s *trans* cleavage. In line with the results obtained upon overexpression of full-length inactive Taspase1 variants, no inhibition of Taspase1’s processing was detectable (**Figure S3a/b**).

To additionally exclude the possibility that the lack of a *trans*-dominant phenotype was restricted to the AF4•MLL protein, we tested the ability of the mutants to interfere with the processing of indicator proteins containing the cleavage-sites from the *bona fide* Taspase1 targets TFIIA (NLS-mCherry/GST-TFIIA_S-NES = TFIIA_S_R_) and USF2 (NLS-mCherry/GST-USF2_S-NES = USF2_S_R_) [7]. No inhibition of processing occurred for these substrates as well as for the full length TFIIA or USF2 proteins (**Table 1**).

### Analysing Taspase1 Heterocomplex-formation

**Figure 4:**
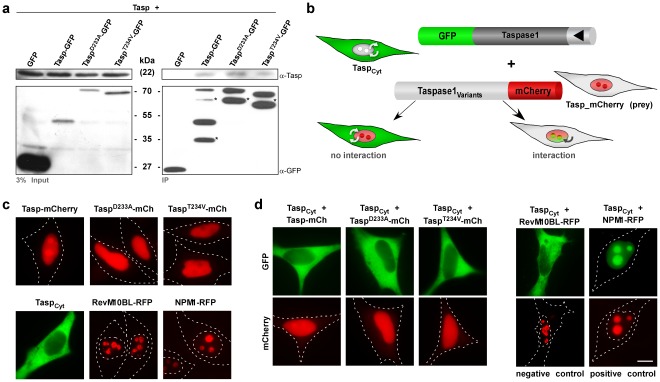
Probing Taspase1 multimerization in living cells. **A.** Heterocomplex formation of Taspase1 and Taspase1 variants shown by co-immunoprecipitation (IP). IPs of 293T cell extracts co-transfected with the indicated expression constructs were carried out using α-GFP Ab-coated magnetic beads and μ-MACS columns. Precipitated proteins were identified by immunoblot using the indicated antibodies. Input: Total amount of cell lysate. IP: immunoprecipitated proteins. *: GFP-degradation products [33]. **B.** Principle of the translocation based protein-protein interaction assay. The Tasp_Cyt_ fusion is composed of GFP, Taspase1 and a NES (?) and thus, continuously shuttling between the nucleus and the cytoplasm. The red-fluorescent Taspase1 variants (Tasp-mCherry prey) accumulate at the nucleus/nucleolus. Upon efficient protein-protein interaction, the GFP-tagged cytoplasmic Tasp_Cyt_ co-localizes with the Tasp-mCherry prey to the nucleus/nucleolus in living cells. **C.** Localization of indicated proteins in the absence of potential interaction partners. **D.** Neither co-expression of WT nor inactive Taspase1 variants resulted in strong nuclear/nucleolar translocation of Tasp_Cyt_. Co-expression of NPM1-RFP, known to strongly interact with Taspase1, triggered nuclear/nucleolar translocation of Tasp_Cyt_ (positive control). In contrast, co-expression of the non-interacting nucleolar RevM10BL-RFP protein showed no effect (negative control) as visualized by fluorescence microscopy in living HeLa transfectants. Scale bars, 10 µm.

In general, interruption of pathobiological relevant protein complexes *via* enforced expression of *trans*-dominant negative mutants critically depends on efficient heterocomplex formation [9], [30]. Thus, the lack of a *trans*-dominant negative effect upon overexpression of inactive Taspase1 mutants may be explained by inefficient heterocomplex formation *in vivo*. Expression of Taspase1-GFP in bacteria showed protein aggregation (**Figure S3c**), which had been previously reported [13]. Co-immunoprecipitation studies of overexpressed Taspase1 and GFP-fusions of the Taspase1 variants also indicated that the WT protein is in principle able to interact with biologically impaired mutants (**Figure 4a**). However, when compared to complex formation of Taspase1 with a *bona fide* interaction partner, the nucleolar protein NPM1, the observed interaction was rather weak (**Figure S3d**) [23].

To further exclude that these results might be valid only for ectopically overexpressed Taspase1, we additionally examined the endogenous protein in MV4;11 human leukemia cells. These cells were isolated from a patient containing a t(4;11) translocation and thus, express the AF4•MLL fusion protein, which is processed by endogenous Taspase1. Employing gel filtration chromatography of cell lysates isolated under native conditions, we detected endogenous Taspase1 predominantly as an αβ-monomer (**Figure S3e**).

### Probing Taspase1 Heterocomplex-formation in Living Cells by a Translocation-based Protein Interaction Assay

Subsequently, we applied a dual color translocation assay that allows visualization of protein complex formation in living cells (**Figure 4b**) to test our hypothesis. This principle has been successfully employed in several studies to assess protein interaction in living cells, including the t(4;11) leukemia relevant MLL-FYRN and -FYRC proteins [9], [22], [23], [31]. Here, GFP-tagged Taspase1 was engineered to localize predominantly to the cytoplasm by C-terminal fusion of a strong nuclear export signal (NES) (Tasp_Cyt_). Due to Taspase1’s intrinsic nuclear import signal, Tasp_Cyt_ is continuously shuttling between the nucleus and the cytoplasm, and still catalytically active (**Figure 4b** **/c**) [23]. The red-fluorescent Taspase1 variants (Tasp-mCherry, prey) however accumulate in the nucleus/nucleolus (**Figure 4c/d**). Upon co-expression and efficient heterocomplex formation, the GFP-tagged Tasp_Cyt_ is expected to co-localize with the Tasp-mCherry prey variants in the nucleus/nucleolus. Therefore, nuclear translocation serves as a reliable indicator for efficient protein-protein interaction in living cells. This approach allows analyzing complex formation between the WT and the inactive mutant enzymes (**Figure 4b**). Co-expression of the positive control, NPM1-RFP, significantly triggered nuclear/nucleolar translocation of GFP-Tasp_Cyt_, whereas co-expression of the non-interacting nucleolar RevM10BL-RFP protein (negative control) showed no effect (**Figure 4d**), confirming the assays specificity. As already expected from the functional data (**Figure 3**), co-expression of mutant Taspase1 variants did not result in strong nuclear/nucleolar translocation of Tasp_Cyt_, indicative of only weak heterocomplex formation (**Figure 4d**). Similar results were obtained upon expression of untagged WT or mutant Taspase1 by immunofluorescence analysis in fixed cells (data not shown). To objectively quantitate the degree of co-localization, we employed confocal laser scanning microscopy revealing a colocalization R-value of 0.74 for NPM1-RFP, 0.19 for RevM10BL-RFP and R-values of 0.38–0.39 for WT and Taspase1 mutants, respectively (**Table S4** and **Figure S4**). Hence, although WT or mutant Taspase1 variants are in principle able to form (hetero)complexes, these complexes appear to be rather unstable.

Subsequently, we investigated whether the individual α- or β-subunits efficiently bound to WT or mutant Taspase1 variants. We found that the nuclear Taspase1_α_-BFP protein (**Figure S5a**, upper picture) was unable to efficiently multimerize with Tasp_Cyt_ and to recruit it to the nucleus (**Figure S5b**). Second, co-expression of Taspase1- or Tasp^T234V^-mCherry did not induce nuclear/nucleolar translocation of Tasp_β_-GFP (**Figure S5a**, lower picture and **S5c**).

Of note, although the subunits were unable to efficiently interact with full length Taspase1, we though observed heterocomplex formation when both subunits were co-expressed. As shown in **Figure S5d**, Tasp_α_-BFP or Tasp_α_-HA recruited Tasp_β_-GFP to the nucleus. Also, an engineered cytoplasmic Tasp-β protein (Tasp-β_Cyt_), accumulated in the nucleus due to complex formation with nuclear Tasp_α_-BFP or Tasp_α_-HA (**Figure S5e**). These results are somehow unexpected as overexpression of the individual Taspase1 α- or β-subunits showed no *trans*-dominant negative effect (**Figure S3c/d**). Thus, we examined whether the complex formed upon coexpression of the individual subunits resembles biologically active Taspase1 generated by autoproteolytic cleavage of the proenzyme. Upon co-expression of Tasp_α_-BFP with Tasp_β_-GFP neither the AF4•MLL- nor the TFIIA-indicator protein was cleaved (**Figure S5f** and data not shown). Similar results were obtained by co-expressing Tasp_α_-HA with untagged Tasp_β_ (data not shown). Hence, co-expression of the individual α- and β-subunit does not allow their assembly into an enzymatically active protease complex. Formation of the αβ-monomer by *cis*-cleavage of the proenzyme seems to occur by a regulated step-wise process.

## Discussion

Recent advances towards the understanding of cancer system biology inspired to consider cancer-related protein-protein interaction networks as potential therapeutic targets [15], [16], [17]. Recently, we used our *in vivo* protein interaction assay to also demonstrate that it is in principle possible to specifically inhibit the AF4•MLL oncoprotein by genetic PPIs inhibitors [9]. As the steady-state amount of the AF4•MLL protein is critically controlled through its processing by Taspase1, it is tempting to target the formation of the active protease by interfering with its multimerization as a novel strategy to block the pathobiological function of AF4•MLL. However, in order to potentially transfer such approaches into the clinics it is imperative to know whether the protein of interest indeed efficiently forms multimers *in vivo* causally required for its pathological functions.

For Taspase1 it is assumed that following autoproteolysis of the zymogen, its subunits assemble into an asymmetric αββα-heterodimer, representing the active protease [6], [13]. This model is mainly based on the crystal structures of other type 2 asparaginases, as well as on the structure obtained from bacterially expressed Taspase1 [13]. Thus, it was concluded that the enzymes consist as a four-layered αββα structure, with a central, mostly anti-parallel β-sandwich that is surrounded by α-helices on both faces [6], [13]. However, experimental evidence convincingly demonstrating that not only Taspase1 but also other type 2 asparaginases do exist in their natural environment as heterodimers, and that multimerization is indeed essential for their biological activities is still missing. Clearly, the structure resolved by Khan et al. provided important insights into Taspase1 function, albeit some limitations may exist [13]. For example, the position of critical functional domains, such as the bipartite NLS can’t be deduced from the current computational model of Taspase1 as these residues are disordered [13], [23]. Also, the structure of the αββα-heterodimer was obtained by co-crystallizing the individual subunits rather than the autoproteolytically processed zymogen. As shown in our study, co-expression of the individual Taspase1 subunits was unable to assemble into a functional protease *in vivo*.

**Figure 5:**
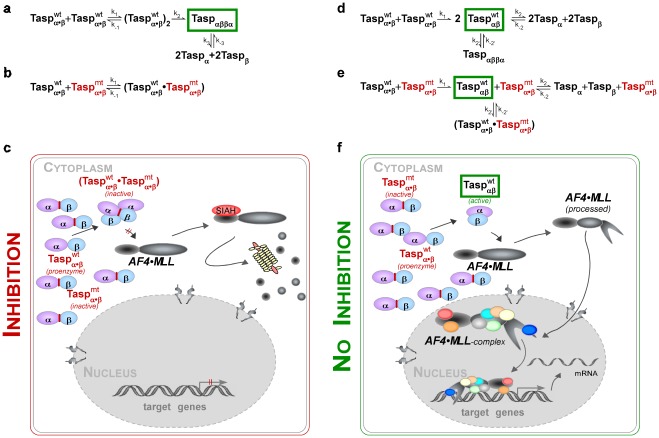
Models illustrating how Taspase1 heterocomplex formation determines the biological effects of overexpressing inactive Taspase1 mutants. A–C: Heterodimer model - allowing inhibition of Taspase1 function by *trans* dominant mutants. **A.** Upon translation, the Taspase1 zymogen dimerizes and following autoproteolysis matures into an asymmetric Taspase1_αββα_-heterodimer, representing the active protease. Taspase1 exist in equilibrium of unprocessed Taspase1 monomers, unprocessed Taspase1 dimers, and active processed Taspase1_αββα_-heterodimers. The Taspase1_αββα_-heterodimers may further dissociate into free Taspase1_α_ and Taspase1_β_ subunits. **B.** Co-expression of an excess of inactive Taspase1 variants results in the formation of catalytically impaired heterodimers, reducing the concentration of active Taspase1 molecules. **C.** Consequently, AF4**•**MLL processing is inhibited allowing its degradation by SIAH1/2, thereby preventing the activation of cellular proliferation programs. **D–F:** Monomer model - predicting Taspase1’s resistance to enforced expression of inactive mutants. **D.** The Taspase1_αβ_ proenzyme is autoproteolytically cleaved, forming an active Taspase1_αβ_ monomer. The processed Taspase1_αβ_ monomer seems to exist also as a Taspase1_αββα_-heterodimer, and potentially in equilibrium with its subunits. **E.** Overexpression of inactive Taspase1 variants does not affect the concentration and activity of Taspase1_αβ_ monomers. **F.** Hence, Taspase1_αβ_ monomers are able to cleave the AF4**•**MLL fusion protein, resulting in the formation of a SIAH-resistant AF4**•**MLL complex allowing the activation of target genes driving oncogenesis.

Based on our data it is thus conceivable to speculate that *in vivo* a complex equilibrium between Taspase1 dimers and already active αβ-monomers might exist (**Figure 5**). According to the “heterodimer model”, the full length Taspase1 zymogen dimerizes, and upon autoproteolysis assembles into an asymmetric Taspase1_αββα_-heterodimer, representing the active protease. Hence, Taspase1 is expected to exist in equilibrium of full length Taspase1 monomers, unprocessed Taspase1 dimers as well as active processed Taspase1_αββα_-heterodimers. The Taspase1_αββα_-heterodimers may further dissociate into free Taspase1_α_ and Taspase1_β_ subunits. The formation of these forms is regulated by their association (k_1_) and dissociation constants (k_–1_) as well as by the kinetics of autoproteolysis, which have not been determined yet (**Figure 5a–c**).

Interruption of pathobiological relevant protein complexes *via* enforced expression of *trans*-dominant negative mutants has been employed in several disease models and requires efficient heterocomplex formation [15], [32]. Assuming that inactive Taspase1 variants are capable of interacting efficiently with the wild type enzyme, a nine-fold overexpression of inactive Taspase1 variants would strongly shift the equilibrium towards the formation of catalytically impaired heterodimers, resulting in a significant *trans*-dominant negative phenotype *in vivo*. For the cases reported, inhibition was already evident upon equimolar co-expression of WT protein and *trans*-dominant mutants, in contrast to what we observed for Taspase1 and inactive Taspase1 variants.

Albeit the current literature does not indicate that tagged- and untagged-Taspase1 behaves differently [7], [23]; this report), we are aware that the results of the *in vivo* protein interaction assays might be affected by the use of Taspase1 variants fused to autofluorescent proteins.

Alternatively, our data could be interpreted by a “monomer model”, in which the Taspase1_αβ_ proenzyme is autoproteolytically cleaved forming a Taspase1_αβ_ monomer, already representing the active protease (**Figure 5d–f**). According to this model, the relative concentrations of these forms and thus, protease activity are regulated by the kinetics of autoproteolysis. As such, it is expected that even an excess of inactive Taspase1 variants would not affect the formation and biological activity of the Taspase1_αβ_ monomers, which is in line with our experimental evidence. Such a model though does not exclude that the cellular pool is composed of Taspase1_αβ_ monomers as well as Taspase1_αββα_-heterodimers. Whether such multimerization has additional biological implications, such as preventing Taspase1 degradation, conferring cleavage-site selectivity or specificity remains to be resolved. Theoretically, processed Taspase1_αβ_ may also exist in equilibrium with its subunits. However our observation that co-expression of the individual α- and β-subunit does not allow their assembly into an enzymatically active protease complex, argues against a highly dynamic exchange of the subunits. Hence, assembly of the αβ-monomer by *cis*-cleavage of the proenzyme seems to occur by an ordered, stepwise process, which might be guided by molecular chaperones, such as NPM1 [23]. In line with our findings, Khan et al. reported significant differences in the structures obtained by co-expression of the individual Taspase1 subunits *versus* the full-length enzyme [13].

As one might argue that the catalytically inactive Tasp^T234V^ variant is unable to achieve a conformation allowing efficient complex formation with active Taspase1, we included the highly attenuated Tasp^D233A^ mutant in the analysis. The molecular mechanism explaining why this mutant showed cleavage-site specificity is not yet resolved. Although this variant thus exists, at least transiently, in a biologically relevant conformation, we did not observe an inhibitory effect.

In conclusion, we provide first evidence that (i) overexpression of biologically inactive Taspase1 mutants as well as of the α- or β-subunits does not interfere with Taspase1’s *cis*- and *trans*-cleavage activity; and (ii) complexes formed by the individual Taspase1 α and β subunit are inactive and do not reflect biologically active Taspase1 generated by autoproteolytic cleavage of the zymogen.

We are aware that besides the models presented here, our results may be explained by alternative models, in which the tetramer only needs one active dimer or has such a high turn over that the effects are not measurable. Clearly, comprehensive experimental work is required to discriminate between these molecular scenarios *in vivo*. However, this would imply that targeting Taspase1 heteromultimerization by genetic or chemical decoys is unlikely to interfere with its (patho)biological activity, including activation of the AF4•MLL oncoprotein (**Figure 5f**). As stabilization of protein complexes by chemical decoys is currently considered an alternative approach to inhibit disease-relevant pathways, it is tempting to investigate such an approach also for Taspase1. Based on our work and studies reported so far, strategies to dissect and selectively inhibit the (patho)biological activity of Taspase1 in oncogenesis are still advised to focus on the (high-throughput) identification of chemicals targeting Taspase1’s catalytic activity.

## Supporting Information

Figure S1 **Optimization of indicator proteins to monitor AF4•MLL processing.** **A.** Nuclear localization of the A•M_S1/2 indicator protein in cancer cell lines expressing high levels of endogenous Taspase1. **B–E.** Optimization of the AF4•MLL cleavage indicator proteins by addition of linker sequences shown in HeLa cells. Integration of the Taspase1 AF4•MLL recognition site alone does not allow processing and nuclear accumulation of the indicator protein by ectopically expressed Taspase1-BFP (**B**) Processing was improved by the integration of a GSGS- (**C**) or KIS-linker (**D**) N-terminal to the cleavage site. The A•M_S2 indicator protein containing the KISQLDGVDDGSGS cleavage site (spacer sequence underlined) showed optimal performance, cytoplasmic in the absence of ectopic Taspase1, whereas co-expression of Taspase1-BFP triggered proteolytic cleavage and complete nuclear translocation (**E**). BFP/GFP-fusion was visualized by fluorescence microscopy in living transfectants. Scale bars, 10 µm. Dashed lines mark cytoplasmic/nuclear cell boundaries obtained from the corresponding phase contrast images.
(PDF)

Figure S2 **Taspase1** ***trans*** **processing of AF4•MLL substrates shows cleavage site-specificity.** **A.** Whereas the indicator protein A•M_S1_R_, containing the first cleavage-site from AF4•MLL, was efficiently processed by Tasp-GFP, both Taspase1 mutants, Tasp^T234V^- or Tasp^D233A^-GFP, were inactive. **B.** In contrast, Tasp^D233A^-GFP was able to partially process A•M_S2_R_, containing the second cleavage-site from AF4•MLL, whereas Tasp^T234V^-GFP was inactive. Proteins were visualized by fluorescence microscopy in living HeLa cell transfected with the indicated expression plasmids 24 h after transfection. Scale bar, 10 µm. **C–D.** Cytoplasmic (C), cytoplasmic and nuclear (N/C) or nuclear (N) fluorescence was counted in at least 200 A•M_S1_R_ (**C**) or A•M_S2_R_ (**D**) -expressing HeLa cell co-transfected with the indicated expression plasmids. Results from a representative experiment are shown. The number of cells displaying cytoplasmic fluorescence significantly decreased upon cotransfection of 0.1 µg Tasp-BFP expression plasmid (***: p<0.0001). Neither Tasp^T234V^- nor Tasp^D233A^-GFP cleaved A•M_S1_R_, but Tasp^D233A^-GFP was able to partially process A•M_S2_R_, containing the second AF4•MLL cleavage-site.
(PDF)

Figure S3 **A.** Expression of GST-Tasp1-GFP (upper panel) in BL21 bacteria shows extensive protein aggregation. In contrast, GST-GFP showed no aggregation (lower panel). Images were taken with identical CCD camera settings. Scale bar, 1 µm. **B.** NPM1 strongly interacts with Tasp-GFP. IPs of 293T cell extracts co-transfected with the indicated expression. Precipitated proteins were identified by immunoblot using the indicated antibodies. Input: Total amount of cell lysate. IP: Immunoprecipitated proteins. **^#^**: GFP-degradation products. **C–D.** Taspase1 *trans*-cleavage is unaffected by overexpression of the Taspase1 α-subunit. HeLa cells were co-transfected with the indicated expression plasmid and analyzed 24 h later. **C.** Even co-transfection of a nine-fold excess of the nuclear Tasp_α_-GFP did not affect A•M_S2_R_ processing and its nuclear translocation. The cleaved red-fluorescent indicator protein, Tasp_α_-GFP, and active Tasp-BFP fusions were independently visualized by fluorescence microscopy in living cells. A representative cell is shown. Scale bar, 10 µm. **D.** The number of cells showing cytoplasmic (C), cytoplasmic and nuclear (N/C) or nuclear (N) fluorescence was counted in at least 200 A•M_S2_R_-expressing cells. Results from a representative experiment are shown. Whereas the number of cell displaying cytoplasmic fluorescence significantly decreased upon co-transfection of 0.1 µg Tasp-BFP expression plasmid, overexpression of Tasp_α_-GFP or GFP alone did not inhibit the activity of Tasp-BFP in *trans*. **E.** Endogenous Taspase1 is detectable predominantly as an αβ-monomer. Cell lysates isolated under native conditions from MV4;11 human leukemia cells were separated by gel filtration chromatography and resolved by 1D-SDS PAGE. Immunoblot analysis of FPLC of MV4;11 cell lysates. Endogenous Taspase1 was visualized in the fractions (49 to 94 kDa) by immunoblot using α-Tasp Ab. *: degradation products.
(PDF)

Figure S4 **Quantitating Taspase1 protein-interaction in living cells by confocal microscopy.** HeLa cells were transfected with the indicated expression plasmids and protein localization as well as co-localization analyzed by confocal microscopy 24 h post transfection. Scale bars, 10 µm. **A–B.** Localization of RevM10BL- (negative control), NPM1-RFP (positive control), Tasp_Cyt_, and the red-fluorescent Taspase1 variants (Tasp_mCherry-prey) in the absence of potential interaction partners in living cells. **C–D.** Quantitation of protein co-localization shown as as scatter gram with the gained Manders overlap coefficient indicated (R values). **C.** Co-expression of RevM10BL-RFP had no effect on Tasp_Cyt_ localization (R = 0.1928), whereas efficient nuclear/nucleolar translocation was observed upon co-expression of NPM1-RFP (R = 0.7354). **D.** In contrast, neither co-expression of WT (R = 0.3867) nor mutant Taspase1 variants (Tasp^D233A^-mCherry, R = 0.3942; Tasp^T234V^-mCherry, R = 0.3876) resulted in strong nuclear/nucleolar translocation of Tasp_Cyt_, indicative of only weak heterocomplex formation in living cells.
(PDF)

Figure S5 **Translocation assay to analyze complex formation of Taspase1 subunits. A–C.** The Taspase1 α- or β-subunits do not form stable heterocomplexes with WT Taspase1. **A.** Localization of Taspase1 α- or β-subunits in HeLa transfectants. Tasp_α_-BFP localizes to the nucleus, whereas Tasp_β_-GFP is nuclear and cytoplasmatic. **B.** Co-expression of Tasp_α_-BFP did not trigger nuclear/nucleolar translocation of full length Tasp_Cyt_. **C.** Also, co-expression of nuclear/nucleolar Tasp-mCh did not translocate Tasp_β_-GFP to the nucleolus. Autofluorescent fusion proteins were visualized in the same cells by fluorescence microscopy. **D–E.** Co-expression of the isolated Taspase1 subunits results in complex formation. **D.** Upon co-expression, nuclear Tasp_α_-BFP associates with Tasp_β_-GFP and recruits to the nucleus. **E.** Also, a cytoplasmatic GFP-Tasp_β_ protein (Tasp-β_Cyt_), generated by fusion of a strong nuclear export signal (left panel), accumulated in the nucleus by binding to nuclear Tasp_α_-BFP (right panel). **F.** Upon co-expression the isolated Taspase1 subunits do not assemble into an enzymatically active protease complex. Co-expression of Tasp_α_-BFP with Tasp_β_-GFP does not result in processing of the A•M_S2_R_ indicator protein. The uncleaved red-fluorescent indicator protein, Tasp_α_-BFP, and Tasp_β_-GFP were independently visualized by fluorescence microscopy in living cells. A representative cell is shown. Scale bar, 10 µm.
(PDF)

Table S1 **List of described disease-associated MLL fusions.** *Abbreviations*: ALL, acute lymphoblastic leukemia; AML, acute myeloid leukemia; CML, chronic myeloid leukemia; JMML, juvenile myelomonocytic leukemia; AUL/ANL, acute undifferentiated leukemia/acute nonlymphocytic leukemia; MDS, myelodysplastic syndromes; tALL/tAML/tMDS, therapy related ALL/AML/MDS; tT-ALL, therapy related T-cell ALL. X: indicates the presence of a putative Taspase1 cleavage site, based on the Taspase1 recognition sequence (Q^3^[F,I,L,V]^2^D^1^↓G^1’^x^2’^D^3’^D^4’^) [1].
(PDF)

Table S2 **List of plasmids used in the study.** Plasmid name, encoded protein, and function are indicated. *: tag used for detection.
(PDF)

Table S3 **Oligonucleotides used for PCR amplification and cloning.** Oligonucleotide name and nucleotide sequence are indicated.
(PDF)

Table S4 **Quantitation of Taspase1 heterocomplex formation by confocal laser scanning microscopy in living cells.** HeLa cells were co-transfected with 1 µg of Tasp_Cyt_ and 1 µg of the indicated mCherry-prey expression plasmids, and analyzed 24 h later. Colocalization coefficients as an indicator of complex formation were calculated using the colocalizer pro software. Rr, Pearson’s correlation coefficient; R, overlap coefficient according to Manders; k1/k2, overlap coefficients; m1/m2, colocalization coefficients. Results from a representative experiment are shown. A colocalization R-value of 0.74 for NPM1-RFP with Tasp_Cyt_ indicates 74% of colocalization.
(PDF)
