# Supplementary figures and images for: Overexpression of the Catalytically Impaired Taspase1T234V or Taspase1D233A Variants Does Not Have a Dominant Negative Effect in T(4;11) Leukemia Cells
Source: PLoS One. 2012 May 3;7(5):e34142. doi: 10.1371/journal.pone.0034142 (PMC3343046; doi:10.1371/journal.pone.0034142)

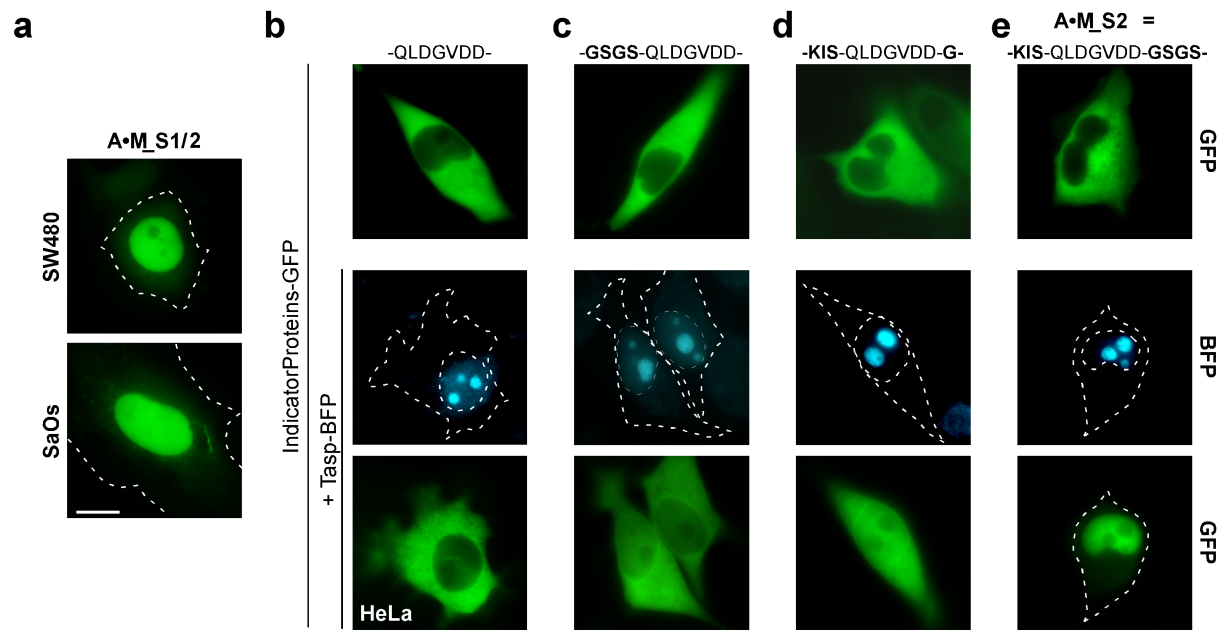

Supplement: Figure S1 — Optimization of indicator proteins to monitor AF4•MLL processing. A. Nuclear localization of the A•M_S1/2 indicator protein in cancer cell lines expressing high levels of endogenous Taspase1. B–E. Optimization of the AF4•MLL cleavage indicator proteins by addition of linker sequences shown in HeLa cells. Integration of the Taspase1 AF4•MLL recognition site alone does not allow processing and nuclear accumulation of the indicator protein by ectopically expressed Taspase1-BFP (B) Processing was improved by the integration of a GSGS- (C) or KIS-linker (D) N-terminal to the cleavage site. The A•M_S2 indicator protein containing the KISQLDGVDDGSGS cleavage site (spacer sequence underlined) showed optimal performance, cytoplasmic in the absence of ectopic Taspase1, whereas co-expression of Taspase1-BFP triggered proteolytic cleavage and complete nuclear translocation (E). BFP/GFP-fusion was visualized by fluorescence microscopy in living transfectants. Scale bars, 10 µm. Dashed lines mark cytoplasmic/nuclear cell boundaries obtained from the corresponding phase contrast images. (PDF) [file pone.0034142.s001.pdf]

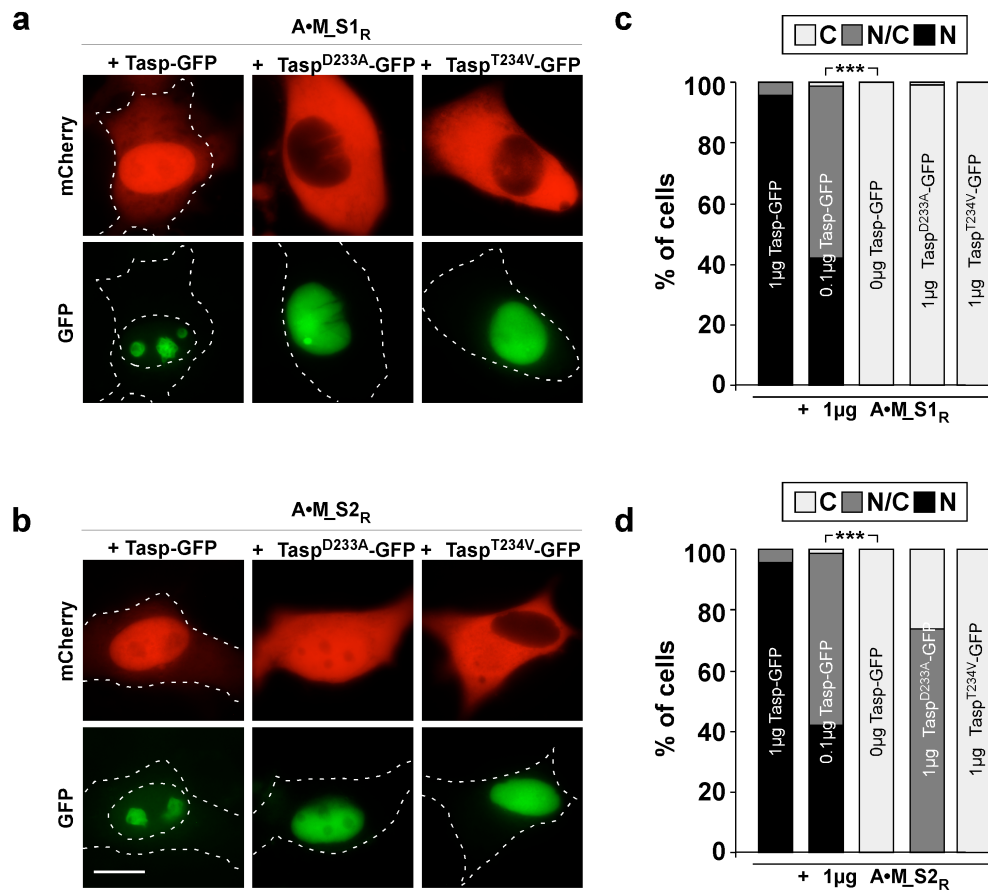

Supplement: Figure S2 — Taspase1 trans processing of AF4•MLL substrates shows cleavage site-specificity. A. Whereas the indicator protein A•M_S1R, containing the first cleavage-site from AF4•MLL, was efficiently processed by Tasp-GFP, both Taspase1 mutants, TaspT234V- or TaspD233A-GFP, were inactive. B. In contrast, TaspD233A-GFP was able to partially process A•M_S2R, containing the second cleavage-site from AF4•MLL, whereas TaspT234V-GFP was inactive. Proteins were visualized by fluorescence microscopy in living HeLa cell transfected with the indicated expression plasmids 24 h after transfection. Scale bar, 10 µm. C–D. Cytoplasmic (C), cytoplasmic and nuclear (N/C) or nuclear (N) fluorescence was counted in at least 200 A•M_S1R (C) or A•M_S2R (D) -expressing HeLa cell co-transfected with the indicated expression plasmids. Results from a representative experiment are shown. The number of cells displaying cytoplasmic fluorescence significantly decreased upon cotransfection of 0.1 µg Tasp-BFP expression plasmid (***: p<0.0001). Neither TaspT234V- nor TaspD233A-GFP cleaved A•M_S1R, but TaspD233A-GFP was able to partially process A•M_S2R, containing the second AF4•MLL cleavage-site. (PDF) [file pone.0034142.s002.pdf]

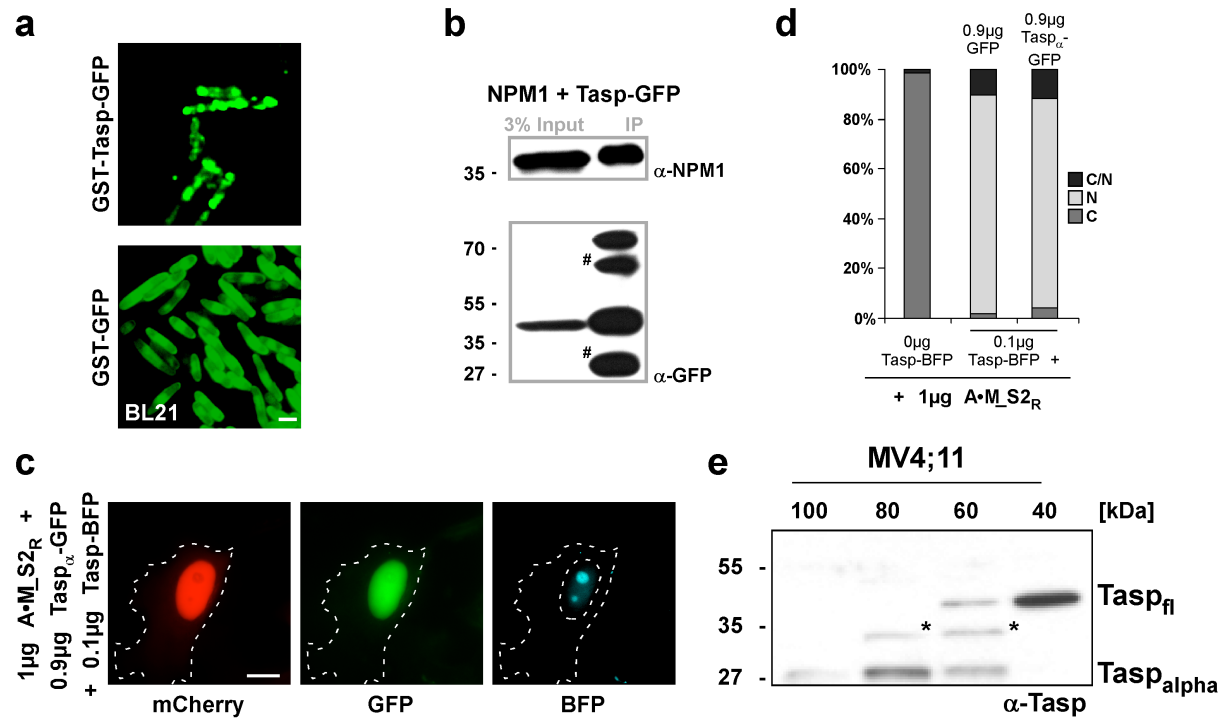

Supplement: Figure S3 — A. Expression of GST-Tasp1-GFP (upper panel) in BL21 bacteria shows extensive protein aggregation. In contrast, GST-GFP showed no aggregation (lower panel). Images were taken with identical CCD camera settings. Scale bar, 1 µm. B. NPM1 strongly interacts with Tasp-GFP. IPs of 293T cell extracts co-transfected with the indicated expression. Precipitated proteins were identified by immunoblot using the indicated antibodies. Input: Total amount of cell lysate. IP: Immunoprecipitated proteins. #: GFP-degradation products. C–D. Taspase1 trans-cleavage is unaffected by overexpression of the Taspase1 α-subunit. HeLa cells were co-transfected with the indicated expression plasmid and analyzed 24 h later. C. Even co-transfection of a nine-fold excess of the nuclear Taspα-GFP did not affect A•M_S2R processing and its nuclear translocation. The cleaved red-fluorescent indicator protein, Taspα-GFP, and active Tasp-BFP fusions were independently visualized by fluorescence microscopy in living cells. A representative cell is shown. Scale bar, 10 µm. D. The number of cells showing cytoplasmic (C), cytoplasmic and nuclear (N/C) or nuclear (N) fluorescence was counted in at least 200 A•M_S2R-expressing cells. Results from a representative experiment are shown. Whereas the number of cell displaying cytoplasmic fluorescence significantly decreased upon co-transfection of 0.1 µg Tasp-BFP expression plasmid, overexpression of Taspα-GFP or GFP alone did not inhibit the activity of Tasp-BFP in trans. E. Endogenous Taspase1 is detectable predominantly as an αβ-monomer. Cell lysates isolated under native conditions from MV4;11 human leukemia cells were separated by gel filtration chromatography and resolved by 1D-SDS PAGE. Immunoblot analysis of FPLC of MV4;11 cell lysates. Endogenous Taspase1 was visualized in the fractions (49 to 94 kDa) by immunoblot using α-Tasp Ab. *: degradation products. (PDF) [file pone.0034142.s003.pdf]

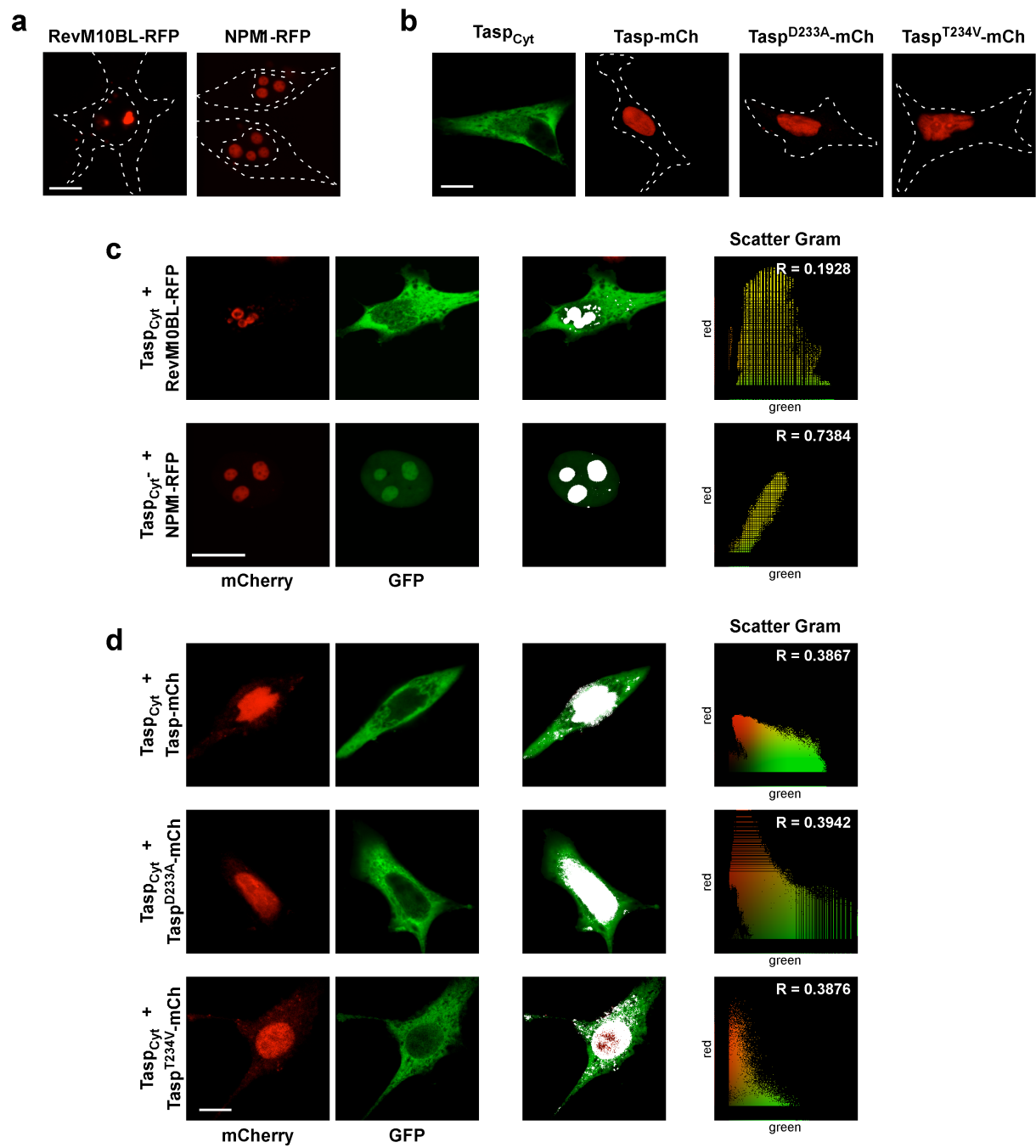

Supplement: Figure S4 — Quantitating Taspase1 protein-interaction in living cells by confocal microscopy. HeLa cells were transfected with the indicated expression plasmids and protein localization as well as co-localization analyzed by confocal microscopy 24 h post transfection. Scale bars, 10 µm. A–B. Localization of RevM10BL- (negative control), NPM1-RFP (positive control), TaspCyt, and the red-fluorescent Taspase1 variants (Tasp_mCherry-prey) in the absence of potential interaction partners in living cells. C–D. Quantitation of protein co-localization shown as as scatter gram with the gained Manders overlap coefficient indicated (R values). C. Co-expression of RevM10BL-RFP had no effect on TaspCyt localization (R = 0.1928), whereas efficient nuclear/nucleolar translocation was observed upon co-expression of NPM1-RFP (R = 0.7354). D. In contrast, neither co-expression of WT (R = 0.3867) nor mutant Taspase1 variants (TaspD233A-mCherry, R = 0.3942; TaspT234V-mCherry, R = 0.3876) resulted in strong nuclear/nucleolar translocation of TaspCyt, indicative of only weak heterocomplex formation in living cells. (PDF) [file pone.0034142.s004.pdf]

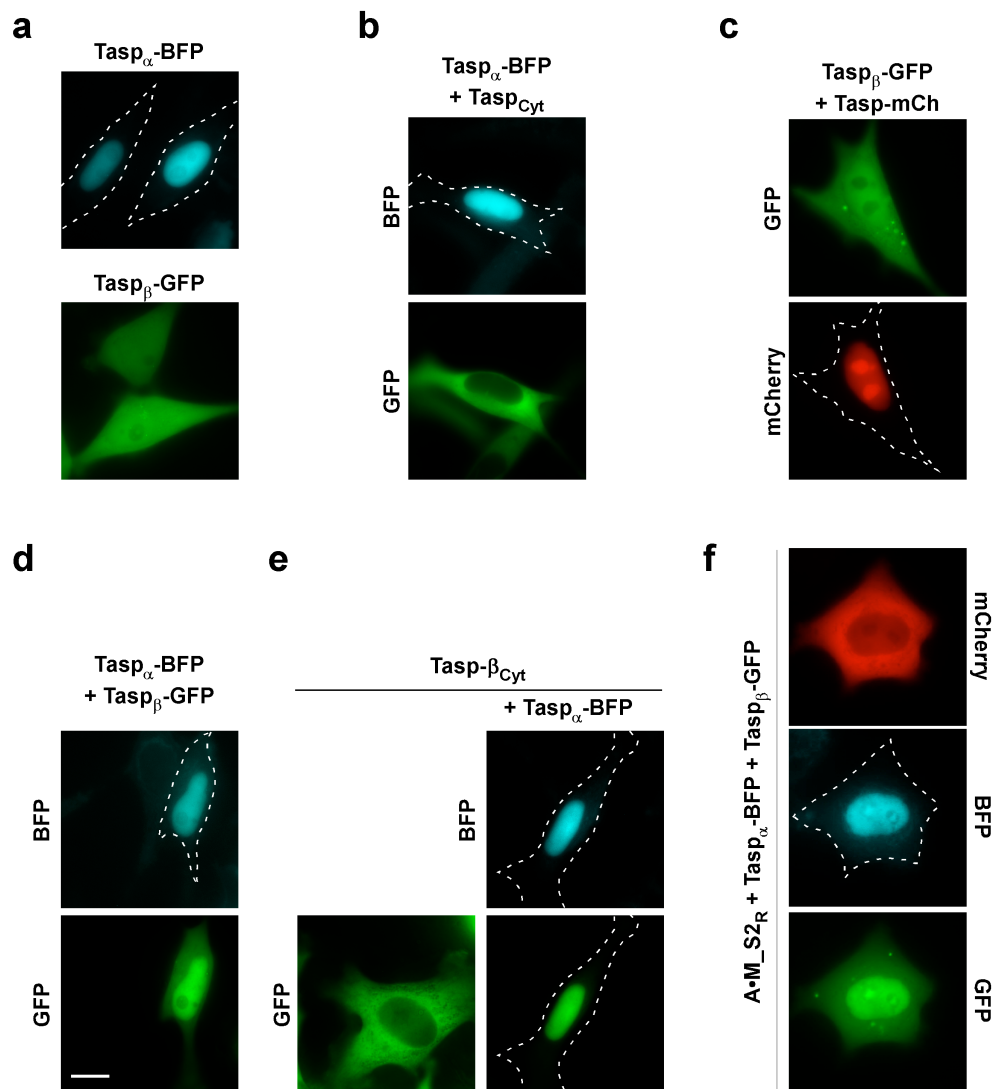

Supplement: Figure S5 — Translocation assay to analyze complex formation of Taspase1 subunits. A–C. The Taspase1 α- or β-subunits do not form stable heterocomplexes with WT Taspase1. A. Localization of Taspase1 α- or β-subunits in HeLa transfectants. Taspα-BFP localizes to the nucleus, whereas Taspβ-GFP is nuclear and cytoplasmatic. B. Co-expression of Taspα-BFP did not trigger nuclear/nucleolar translocation of full length TaspCyt. C. Also, co-expression of nuclear/nucleolar Tasp-mCh did not translocate Taspβ-GFP to the nucleolus. Autofluorescent fusion proteins were visualized in the same cells by fluorescence microscopy. D–E. Co-expression of the isolated Taspase1 subunits results in complex formation. D. Upon co-expression, nuclear Taspα-BFP associates with Taspβ-GFP and recruits to the nucleus. E. Also, a cytoplasmatic GFP-Taspβ protein (Tasp-βCyt), generated by fusion of a strong nuclear export signal (left panel), accumulated in the nucleus by binding to nuclear Taspα-BFP (right panel). F. Upon co-expression the isolated Taspase1 subunits do not assemble into an enzymatically active protease complex. Co-expression of Taspα-BFP with Taspβ-GFP does not result in processing of the A•M_S2R indicator protein. The uncleaved red-fluorescent indicator protein, Taspα-BFP, and Taspβ-GFP were independently visualized by fluorescence microscopy in living cells. A representative cell is shown. Scale bar, 10 µm. (PDF) [file pone.0034142.s005.pdf]
