# Supplementary material for: Overexpression of the Catalytically Impaired Taspase1T234V or Taspase1D233A Variants Does Not Have a Dominant Negative Effect in T(4;11) Leukemia Cells
Source: PLoS One. 2012 May 3;7(5):e34142. doi: 10.1371/journal.pone.0034142 (PMC3343046; doi:10.1371/journal.pone.0034142)
Supplement: Table S1 — List of described disease-associated MLL fusions. Abbreviations: ALL, acute lymphoblastic leukemia; AML, acute myeloid leukemia; CML, chronic myeloid leukemia; JMML, juvenile myelomonocytic leukemia; AUL/ANL, acute undifferentiated leukemia/acute nonlymphocytic leukemia; MDS, myelodysplastic syndromes; tALL/tAML/tMDS, therapy related ALL/AML/MDS; tT-ALL, therapy related T-cell ALL. X: indicates the presence of a putative Taspase1 cleavage site, based on the Taspase1 recognition sequence (Q3[F,I,L,V]2D1↓G1’x2’D3’D4’) [1]. (PDF) [file pone.0034142.s006.pdf]

Supplementary Table S1 – List of described disease-associated MLL fusions.

fusion partner	translocation	provoked leukemia	putative Taspase1 cleavage
AFF1 (AF4)	t(4;11)(q21;q23)	ALL, tAML, AML, tALL	x
T4 (AF6)	t(6;11)(q27;q23)	AML, tAML, ALL	x
T3 (AF9)	t(9;11) (p22;q23)	AML, tAML, ALL	x
T10 (AF10)	t(10;11)(p12;q23)	AML, tAML, ALL	x
ELL	t(11;19)(q23;p13.1)	AML, tAML	
T1 (ENL)	t(11;19)(q23;p13.3)	ALL, AML, tALL	x
AFX1	t(X;11)(q13;q23)	tALL, ALL, AML	x
Septin2	t(X;11)(q22;q23)	AML	x
EB15 (AF1p)	t(1;11)(p32;q23)	ALL, AML, CML	
T11 (AF1q)	t(1;11)(q21;q23)	AML	
AFF3 (LAF4)	t(2;11)(q11;q23)	ALL	x
NCKIPSD (AF3p21)	t(3;11)(p21;q23)	ALL, tAML	x
EEFSEC (SELB)	t(3;11)(q21;q23)	ALL	x
GMPS	t(3;11)(q25;q23)	tAML	
LPP	t(3;11)(q28;q23)	tAML	x
AF4p12	t(4;11)(p12;q23)	tALL, tAML	x
MIFL	t(4;11)(q12;q23)	tAML, tALL	
SEPT11	t(4;11)(q21;q23)	atypical CML	x
ArgBP2	t(4;11)(q35;q23)	AML	
ARHGAP26 (GRAF)	t(5;11)(q31;q23)	ALL, JMML	x
AFF4 (AF5q31)	ins(5;11)(q31;q13q23)	ALL	
SMAP1	t(6;11)(q12;q23)	AML	x
FOXO3A (AF6q21)	t(6;11)(q21;q23)	tAML	x
DAB2IP (AF9q34)	t(9;11)(q34;q23)	AML	x
ABI1	t(10;11)(p11;q23)	AML	x
CXXC6 (TET1)	t(10;11)(q21;q23)	AML	x
PICALM	t(11;11)(q21;q23)	AML	x
tandem duplication	trisomy 11	AML	x
CBL	t(11;11)(q23;q23)	AML	
ARHGEF12 (LARG)	t(11;11)(q23;q23)	AML	x
TIRAP	t(11;11)(q23;q24)	AML	x
CIP29	t(11;12)(q23;q13)	AUL/ANL, AML	x
GPHN	t(11;14)(q23;q24)	AML, tAML	x
CASC5 (AF15q14)	t(11;15)(q23;q14)	ALL, AML	
MPFYVE	t(11;15)(q23;q14)	AML	x
AF15	t(11;15)(q23;q15)	AML	
CREBBP (CBP)	t(11;16)(q23;p13)	MDS, ALL, tAML, tALL, tCML, tMDS	x

GAS7	t(11;17)(q23;p13)	tAML	
ACACA	t(11;17)(q23;q21)	AML	x
LASP1	t(11;17)(q23;q21)	AML	x
T6 (AF17)	t(11;17)(q23;q21)	AML	x
RARa	t(11;17)(q23;q21)	AML	
SEPT9 (MSF1, AF17q25)	t(11;17)(q23;q25)	MDS, tAML, AML	x
SH3GLI1 (EEN)	t(11;19)(q23;p13)	AML	
MYO1F	t(11;19)(q23;p13)	AML	x
MAPRE1 (EB1)	t(11;20)(q23;q11)	ALL	x
SEPT5 (hCDCRel)	t(11;22)(q23;q11.2)	AML, tALL	
EP300 (P300)	t(11;22)(q23;q13)	tAML	
Senp6	Ins(X;11)(q24;q23)	AML	
CDK6	t(4;7;11)(q21;q21 22;q23)	ALL, AML	x
CXXC6	t(10;11)(q21;q23)	AML	x
RPS3	Del(11)(q13.3q23)	AML	
SEPT2	t(2;11)(q37;q23)	AML, tMDS, tAML	
DCP1A	t3;11)(p21.3;q23)	ALL	
CENPK/FKSG14	*complex abnormalities*	AML	
TNRC18/KIAA1856	t(7;11)(p22.1;q23)	ALL	
FNBP1/FBP17	Ins(11;9)(q23;q34)inv(11)(q13q23)	AML	x
LAMC3	t(9;11)(q31~q34;q23)	tAML	
NEBL	Ins(10;11)(p12;q23)	AML	x
NRIP3	Inv(11)(p15.3q23)	AML	
ARHGEF17	t(11;11)(q13.4;q23)	AML	x
C2CD3/DKFZP586P0123	Inv(11)(q13.4q23)	AML	
MAML2	Inv(11)(q21q23)	tT-ALL, tAML	x
UBE4A	t(11;15)(q23q;q21)inv(11)(q23q23)	MDS	
BCL9L	Del(11)(q23q23.3)	ALL	
DCPS	Del(11)(q23q24.2)	AML	
KIAA0284	t(11;14)(q32.33;q32.33)	AML	
VAV1	Ins(11;19)(q23;p13.2)	AML	
ASAH3/ACER1	t(11;19)(q23;p13.3)	ALL	
LOC100128568	t(2;11;19)(p23.3;q23;p13.3)	AML	
ACTN4	t(11;19)(q23;q13)	ALL	x
FLNA	Ins(11;X)(q23;q28q13.1)	AML	

Abbreviations: ALL, acute lymphoblastic leukemia; AML, acute myeloid leukemia; CML, chronic myeloid leukemia; JMML, juvenile myelomonocytic leukemia; AUL/ANL, acute undifferentiated leukemia/ acute nonlymphocytic leukemia; MDS, myelodysplastic syndromes; tALL/tAML/tMDS, therapy related ALL/AML/MDS; tT-ALL, therapy related T-cell ALL. **X**: indicates the presence of a putative Taspase1 cleavage site, based on the Taspase1 recognition sequence ($Q^3[F,I,L,V]^2D^1{\downarrow}G^{1'}x^{2'}D^{3'}D^{4'}$).[1]
