# Supplementary material for: Overexpression of the Catalytically Impaired Taspase1T234V or Taspase1D233A Variants Does Not Have a Dominant Negative Effect in T(4;11) Leukemia Cells
Source: PLoS One. 2012 May 3;7(5):e34142. doi: 10.1371/journal.pone.0034142 (PMC3343046; doi:10.1371/journal.pone.0034142)
Supplement: Table S2 — List of plasmids used in the study. Plasmid name, encoded protein, and function are indicated. *: tag used for detection. (PDF) [file pone.0034142.s007.pdf]

Supplementary Table S2 – List of plasmids used in the study.

Plasmid	encoding	tag*	function
eukaryotic expression			
pTasp	wt Taspase1	untagged	protease
pTasp-GFP	wt Taspase1	GFP	protease
pTasp-BFP	wt Taspase1	BFP	protease
pTasp-mCh	wt Taspase1	mCherry	protease
pTasp-HA	wt Taspase1	HA-tag	protease
pTasp^{T234V}	inactive Taspase1 mutant; Thr^{234} → Val	untagged	inactive protease
pTasp^{T234V}-GFP	inactive Taspase1 mutant; Thr^{234} → Val	GFP	inactive protease
pTasp^{T234V}-BFP	inactive Taspase1 mutant; Thr^{234} → Val	BFP	inactive protease
pTasp^{T234V}-mCh	inactive Taspase1 mutant; Thr^{234} → Val	mCherry	inactive protease
pTasp^{T234V}-HA	inactive Taspase1 mutant; Thr^{234} → Val	HA-tag	inactive protease
pTasp^{D233A}	attenuated Taspase1 mutant; Asp^{233} → Ala	untagged	attenuated protease
pTasp^{D233A}-GFP	attenuated Taspase1 mutant; Asp^{233} → Ala	GFP	attenuated protease
pTasp^{D233A}-BFP	attenuated Taspase1 mutant; Asp^{233} → Ala	BFP	attenuated protease
pTasp^{D233A}-mCh	attenuated Taspase1 mutant; Asp^{233} → Ala	mCherry	attenuated protease
pTasp^{D233A}-HA	attenuated Taspase1 mutant; Asp^{233} → Ala	HA-tag	attenuated protease
pTasp$_{\alpha}$	Taspase1, α-subunit	untagged	protease subunit
pTasp$_{\alpha}$-GFP	Taspase1, α-subunit	GFP	protease subunit
pTasp$_{\alpha}$-BFP	Taspase1, α-subunit	BFP	protease subunit
pTasp$_{\alpha}$-HA	Taspase1, α-subunit	HA-tag	protease subunit
pTasp$_{\beta}$	Taspase1, β-subunit	untagged	protease subunit
pTasp$_{\beta}$-GFP	Taspase1, β-subunit	GFP	protease subunit
pTasp$_{Cyt}$	GFP-Tasp-NES$_{Rev}$;	GFP,<br>N-term	cytoplasmic<br>Taspase1
pTasp-β$_{Cyt}$	GFP-Tasp$_{\beta}$-NES$_{Rev}$	GFP,<br>N-term	cytoplasmic<br>Taspase1 β-subunit
pA•M_S1/2	NLS-GFP/GST-AF4•MLL_S1/2-NES$_{Rev}$;<br>S1/2: both AF4•MLL cleavage sites aa 1582-1710; S1: ^{1600}AEGQVDGADD^{1609};<br>S2: ^{1652}KISQLDGVDD^{1661}	GFP	AF4•MLL<br>cleavage sensor
pA•M_S1	NLS-GFP/GST-AF4•MLL_S1-NES$_{Rev}$;<br>S1: ^{1600}AEGQVDGADD^{1609}	GFP	AF4•MLL<br>S1 cleavage sensor
pA•M_S1$_{mut}$	NLS-GFP/GST-AF4•MLL_S1$_{mut}$-NES$_{Rev}$;<br>S1$_{mut}$: ^{1600}AEGQVAAADD^{1609}	GFP	unfunctional<br>AF4•MLL<br>S1 cleavage sensor
pA•M_S2	NLS-GFP/GST-AF4•MLL_S2-NES$_{Rev}$;<br>S2: ^{1652}KISQLDGVDD^{1661}	GFP	AF4•MLL<br>S2 cleavage sensor
pA•M_S2$_{mut}$	NLS-GFP/GST-AF4•MLL_S2$_{mut}$-NES$_{Rev}$;<br>S2$_{mut}$: ^{1652}KISQLAAVDD^{1661}	GFP	unfunctional S2<br>cleavage sensor
pA•M_S1$_{R}$	NLS-mCh/GST-AF4•MLL_S1-NES$_{Rev}$;<br>S1: ^{1600}AEGQVDGADD^{1609}	mCherry	AF4•MLL<br>S1 cleavage sensor
pA•M_S2$_{R}$	NLS-mCh/GST-AF4•MLL_S2-NES$_{Rev}$;<br>S2: ^{1652}KISQLDGVDD^{1661}	mCherry	AF4•MLL<br>S2 cleavage sensor
pS_QLDGVDD	NLS-GFP/GST-QLDGVDD-NES$_{Rev}$;<br>^{1655}QLDGVDD^{1661}	GFP	constricted<br>AF4•MLL<br>cleavage sensor

pS_GSGS-QLDGVDD	NLS-GFP/GST-GSGS-QLDGVDD-NES_{Rev}; GSGS-linker + ^{1655}QLDGVDD^{1661}	GFP	constricted AF4•MLL cleavage sensor
pS_KIS-QLDGVDD-G	NLS-GFP/GST-KIS-QLDGVDD-G-NES_{Rev}; ^{1652}KISQLDGVDD^{1661} + G-linker	GFP	constricted AF4•MLL cleavage sensor
pTFIIA_S_R	NLS-mCh/GST-TFIIA-NES_{Rev}	mCherry	TFIIA cleavage sensor
pUSF2_S_R	NLS-mCh/GST-USF2-NES_{Rev}	mCherry	USF2 cleavage sensor
pCasp3-Clev	NLS-GFP/GST-CS3-NES_{Rev}; CS3: ^{207}KRKGDEVDGVDE^{218}	GFP	Caspase3 cleavage sensor
pTFIIA-GFP	wt TFIIA	GFP	Taspase1 substrate
pUSF2-GFP	wt USF2	GFP	Taspase1 substrate
pNPM1	wt Nucleophosmin	untagged	nucleolar Taspase1 interaction partner
pNPM1-RFP	wt Nucleophosmin	RFP	nucleolar Taspase1 interaction partner
pRevM10BL-BFP	export-deficient mutant of HIV-1 Rev	BFP	nucleolar protein (- control)
pRevM10BL-RFP	export-deficient mutant of HIV-1 Rev	RFP	nucleolar protein (- control)
pGFP = pF143	GFP	GFP	control
pBFP = F145	BFP	BFP	control
pBluescript (pBSK)	vector backbone	untagged	control
prokaryotic expression			
pGEX_GST-Tasp-GFP	GST-Tasp-GFP; wt Taspase1	GFP	protease
pGEX_GST-GFP	GST-GFP	GFP	control

Plasmid name, encoded protein, and function are indicated. *: tag used for detection.
