# Supplementary material for: Overexpression of the Catalytically Impaired Taspase1T234V or Taspase1D233A Variants Does Not Have a Dominant Negative Effect in T(4;11) Leukemia Cells
Source: PLoS One. 2012 May 3;7(5):e34142. doi: 10.1371/journal.pone.0034142 (PMC3343046; doi:10.1371/journal.pone.0034142)
Supplement: Table S3 — Oligonucleotides used for PCR amplification and cloning. Oligonucleotide name and nucleotide sequence are indicated. (PDF) [file pone.0034142.s008.pdf]

Supplementary Table S3 - Oligonucleotides used for PCR amplification and cloning.

Primer/Oligo	Sequence
Taspase_XhoI*5	5'-CCGCTCGAGGTTCACTGGGCTCTCCA-3'
Taspase_BamHI*3	5'-TTTGGATCCACATGACCATGGAGAAGGGGATG-3'
Taspase_NheI*5	5'-AAAGCTAGCGTTCACTGGGCTCTCCAGGCGG-3'
Taspase-stop_EcoRI*5	5'-TTTGAATTCTTAGTTCACTGGGCTCTCCAGGCGG-3'
Taspase_EcoRI_SOE*5	5'-CGGCTGGCAGAATACCTCCCTGC-3'
Taspase_EcoRI_SOE*3	5'-GCAGGGAGGTATTCTGCCAGCCG-3'
TaspaseD233A_SOE*5	5'-GGCACTTTGGCCACGGTAGGC-3'
TaspaseD233A_SOE*3	5'-GCCTACCGTGGCCAAAGTGCC-3'
TaspaseT234V_SOE*5	5'-CACTTTGGACGTGGTAGGCGCTG-3'
TaspaseT234V_SOE*3	5'-CAGCGCCTACCACGTCCAAAGTG-3'
Taspase-beta_Not*5	5'-TTTGCGGCCGCAACGGTAGGCGCTGTGGTTGTGGAC-3'
Taspase_XhoI*3	5'-AAACTCGAGCGTTCACTGGGCTCTCCAGGCGGCAC-3'
Taspase_NotI*5	5'-TTTGCGGCCGCAATGACCATGGAGAAGGGGATGAG-3'
Taspase-alpha_XhoI*3	5'-AAACTCGAGCGTCCAAAGTGCCTGAGTCATTTTC-3'
Taspase_KpnI*5	5'-TTTGGTACCATGACCATGGAGAAGGGGATGAGTTC-3'
Taspase-beta_BamHI*5	5'-TTTGGATCCATGACGGTAGGCGCTGTGGTTGTG-3'
Taspase-alpha_NheI*3	5'-TTTGCTAGCGTCCAAAGTGCCTGAGTC-3'
mCherry-NheI*3	5'-TTTGCTAGCAAGGGCGAGGAGGATAAC-3'
mCherry_Stop-EcoRI*5	5'-TTTGAATTCTTACTTGTACAGCTCGTCC-3'
mCherry_KpnI*5	5'-TTTGGTACCCTTGTACAGCTCGTCCATGC-3'
AF4•MLL-S1/2_NotI*5	5'-AAAGCGGCCGCGCGTTCTAACATGTTTTTTGG-3'
AF4•MLL-S1/2_XhoI*3	5'-AAACTCGAGAACTCTTAGTGACATGTTCTTTC-3'
S2_NotI*5	5'-GGCCGCACAGCTTGATGGTGTTGATGATGGCGC-3'
S2_XhoI*3	5'-TCGAGCGCCATCATCAACACCATCAAGCTGTGC-3'
GSGS-S2_XhoI*3	5'-TCGAGCATCGCTCTCCGTGCCATCATCAACACCATCAAGCTGGCTGCCAGAGC CTGC-3'
GSGS-S2_NotI*5	5'-GGCCGCAGGCTCTGGCAGCCAGCTTGATGGTGTTGATGATGGCACGGAGAGC GATGC-3'
KIS-S2-DG_NotI*5	5'-GGCCGCAAAAATCAGCCAGCTTGATGGTGTTGATGATGGCACGGAGAGCGGT GC-3'
KIS-S2-DG_XhoI*3	5'-TCGAGCACCGCTCTCCGTGCCATCATCAACACCATCAAGCTGGCTGATTTTTG C-3'
AF4•MLL-S2_NotI*5	5'-GGCCGCAAAAATCAGCCAGCTTGATGGTGTTGATGATGGCTCTGGCAGCGC-3'
AF4•MLL-S2_XhoI*3	5'-TCGAGCGCTGCCAGAGCCATCATCAACACCATCAAGCTGGCTGATTTTTGC-3'
AF4•MLL-S1_NotI*5	5'-GGCCGCAGCTGAAGGACAGGTGGATGGGGCCGATGACTTAAGCACTTCAGC-3'
AF4•MLL-S1_XhoI*3	5'-TCGAGCTGAAGTGCTTAAGTCATCGGCCCCATCCACCTGTCCTTCAGCTGC-3'
NPM1_BamHI*5	5'-TTTGGATCCACATGGAAGATTCGATGGACATGGAC-3'
NPM1_NheI*3	5'-TTTGCTAGCTCCTGCACCAAGAGACTTCCTCC-3'
NPM1-stop_EcoRI*3	5'-TTTGAATTCAAAGAGACTTCCTCCACTGCCAG-3'

Oligonucleotide name and nucleotide sequence are indicated.
