# Supplementary material for: Overexpression of the Catalytically Impaired Taspase1T234V or Taspase1D233A Variants Does Not Have a Dominant Negative Effect in T(4;11) Leukemia Cells
Source: PLoS One. 2012 May 3;7(5):e34142. doi: 10.1371/journal.pone.0034142 (PMC3343046; doi:10.1371/journal.pone.0034142)
Supplement: Table S4 — Quantitation of Taspase1 heterocomplex formation by confocal laser scanning microscopy in living cells. HeLa cells were co-transfected with 1 µg of TaspCyt and 1 µg of the indicated mCherry-prey expression plasmids, and analyzed 24 h later. Colocalization coefficients as an indicator of complex formation were calculated using the colocalizer pro software. Rr, Pearson’s correlation coefficient; R, overlap coefficient according to Manders; k1/k2, overlap coefficients; m1/m2, colocalization coefficients. Results from a representative experiment are shown. A colocalization R-value of 0.74 for NPM1-RFP with TaspCyt indicates 74% of colocalization. (PDF) [file pone.0034142.s009.pdf]

**Supplementary Table S4 - Quantitation of Taspase1 heterocomplex formation by confocal laser scanning microscopy in living cells.**

| $Tasp_{Cyt}$+ | coefficient | | | | | |
|---|---|---|---|---|---|---|
| mCherry prey | Rr | R | k1 | k2 | m1 | m2 |
| Tasp-mCh | -0.3926 | 0.3867 | 0.7246 | 0.2063 | 0.9902 | 0.3494 |
| $Tasp^{D233A}$-mCh | -0.1993 | 0.3942 | 0.5365 | 0.2896 | 1.0000 | 0.4104 |
| $Tasp^{T234V}$-mCh | -0.3323 | 0.3876 | 0.4679 | 0.3211 | 0.8808 | 0.3676 |
| NPM1-RFP | 0.9490 | 0.7384 | 0.8336 | 0.6541 | 0.9998 | 0.3866 |
| RevM10BL-RFP | -0.1535 | 0.1928 | 0.5977 | 0.0622 | 0.8431 | 0.0854 |

HeLa cells were co-transfected with 1µg of $Tasp_{Cyt}$ and 1µg of the indicated mCherry-prey expression plasmids, and analyzed 24h later. Colocalization coefficients as an indicator of complex formation were calculated using the colocalizer pro software. Rr, Pearson's correlation coefficient; R, overlap coefficient according to Manders; k1/k2, overlap coefficients; m1/m2, colocalization coefficients. Results from a representative experiment are shown. A colocalization R-value of 0.74 for NPM1-RFP with $Tasp_{Cyt}$ indicates 74% of colocalization.
